# Tissue-specific variation in DNA methylation levels along human chromosome 1

**DOI:** 10.1186/1756-8935-2-7

**Published:** 2009-06-08

**Authors:** Cecilia De Bustos, Edward Ramos, Janet M Young, Robert K Tran, Uwe Menzel, Cordelia F Langford, Evan E Eichler, Li Hsu, Steve Henikoff, Jan P Dumanski, Barbara J Trask

**Affiliations:** 1 Department of Genetics and Pathology, Rudbeck Laboratory, Uppsala University, Uppsala, Sweden; 2 Division of Human Biology, Fred Hutchinson Cancer Research Center, Seattle, Washington, USA; 3 Department of Genome Sciences, University of Washington, Seattle, Washington, USA; 4 Division of Basic Sciences, Fred Hutchinson Cancer Research Center, Seattle, Washington, USA; 5 The Wellcome Trust Sanger Institute, Wellcome Trust Genome Campus, Hinxton, Cambridge, UK; 6 Howard Hughes Medical Institute, Seattle, Washington, USA; 7 Division of Public Health Sciences, Fred Hutchinson Cancer Research Center, Seattle, Washington, USA; 8 Current address: United Nations World Food Programme, Lima, Peru; 9 Current address: National Institutes of Health, Bethesda Maryland, USA; 10 Current address: Genome Center, University of California at Davis, Davis, California, USA

## Abstract

**Background:**

DNA methylation is a major epigenetic modification important for regulating gene expression and suppressing spurious transcription. Most methods to scan the genome in different tissues for differentially methylated sites have focused on the methylation of CpGs in CpG islands, which are concentrations of CpGs often associated with gene promoters.

**Results:**

Here, we use a methylation profiling strategy that is predominantly responsive to methylation differences outside of CpG islands. The method compares the yield from two samples of size-selected fragments generated by a methylation-sensitive restriction enzyme. We then profile nine different normal tissues from two human donors relative to spleen using a custom array of genomic clones covering the euchromatic portion of human chromosome 1 and representing 8% of the human genome. We observe gross regional differences in methylation states across chromosome 1 between tissues from the same individual, with the most striking differences detected in the comparison of cerebellum and spleen. Profiles of the same tissue from different donors are strikingly similar, as are the profiles of different lobes of the brain. Comparing our results with published gene expression levels, we find that clones exhibiting extreme ratios reflecting low relative methylation are statistically enriched for genes with high expression ratios, and *vice versa*, in most pairs of tissues examined.

**Conclusion:**

The varied patterns of methylation differences detected between tissues by our methylation profiling method reinforce the potential functional significance of regional differences in methylation levels outside of CpG islands.

## Background

DNA methylation is a major epigenetic modification that is vital to mammalian development [1]. Methylation is carried out by DNA methyltransferases [2] and can suppress the initiation of transcription of a locus [3]. Methylation plays a significant role in genomic imprinting, X-inactivation, and silencing of parasitic sequences [4-7]. Acquired DNA methylation differences might account for some phenotypic differences between monozygotic twins [8]. Aberrant methylation can cause various syndromes [9-14] and contribute to tumorigenesis by decreasing activity of tumor suppressor genes [15], activating proto-oncogenes [16], or overall methylation imbalance [17] (reviewed in [18]).

In mammals, methylation occurs preferentially at cytosines that are followed by guanine (CpG). CpG dinucleotides are relatively infrequent in the human genome, except in CpG islands, which are small (0.2 to 2 kb) regions highly enriched in CpGs. Approximately 50 to 60% of gene promoters lie in CpG islands [19-21]. Methylation can inhibit binding of transcription factors to these sequences directly by altering the structure of the recognition site, or indirectly by recruiting repressive proteins with methyl-binding domains [22-25].

However, methylation of CpG island promoters does not always correlate with gene expression levels. Most inactive promoters are not methylated [3,26]. The CpG islands of some tissue-specific genes remain unmethylated in tissues where the gene is not expressed (such as *MyoD *in non-muscle tissues) [27-29]. Intriguingly, while promoters are less methylated on the active X than on the inactive X, the opposite is true for CpGs in the bodies of genes on the X chromosome [30]. Highly expressed autosomal genes were also found to have hypomethylated promoters and hypermethylated gene bodies in a recent genome-wide analysis [31]. Moreover, clusters of hypomethylated regions not limited to CpG islands or promoters have been found interspersed in stretches of methylated CpGs [32]. CpG methylation outside of CpG islands is thought to suppress transcription of transposable elements and other parasitic sequences [26,33] and/or to suppress spurious initiation of transcription elsewhere, including within infrequently transcribed genes [34,35]. Clearly more information on the state of methylation in and outside of CpG islands and across different tissues is needed to clarify the role methylation plays in transcriptional regulation.

Several approaches have been developed recently to scan genomes for sites of DNA methylation that might be important for tissue-specific gene regulation. These approaches include large-scale sampling of DNA sequences after bisulfite conversion of unmethylated cytosines to uracil [31,33,36-38], a variety of techniques measuring the differential sensitivity of methylated and unmethylated sequences to certain restriction enzymes (for example, RLGS, RDA, HELP, MCAM, and MSCC) [31,32,39-48], and quantitative analyses of promoter sequences immunoprecipitated with antibodies that enrich for methylated cytosines [26,49,50] (reviewed in [51]). Differences in methylation have been detected among somatic tissues at up to 15% of the loci analyzed in these studies [26,31,33,36,45,48,52].

Here, we employ a methylation profiling technique and a tiling microarray for human chromosome 1 to detect regional differences in the methylation state between tissues from the same individual. Our approach relies on a methylation-sensitive restriction enzyme to generate differently sized fragments from methylated *versus *non-methylated genomic DNA. Sample differences in fragment yield within a specified size range (80 to 2,500 bp) are detected by competitive hybridization of size-selected fragments to a tiling array of genomic clones. This approach has been used successfully to characterize methylation patterns in *Arabidopsis *methylation mutants [53,54]. We have combined this technique with a custom chromosome 1 microarray with a resolution of approximately 100 kb [55].

Below, we first describe simulation experiments that show that log ratios measured by the array are a complex function of the methylation level of the hybridizing DNA and the GC-content of the array clones. We also predict from simulations that the array will be more responsive to methylation changes at dispersed CpG sites than to changes in CpG islands. We then use the array to profile chromosome 1 methylation in several tissues from two human donors, demonstrating that the technique yields highly reproducible results. The methylation profiles we obtain vary between different human tissues, but profiles of the same tissue from two different individuals are similar. We then describe our use of bisulfite sequencing to confirm an array finding of hypomethylation in heart DNA around the *RYR2 *gene and to confirm predictions from our simulations that observed log ratios are impacted by methylation differences outside of CpG islands. We analyze published data on the relative expression levels of genes and detect significantly different expression ratios for genes residing in regions exhibiting extremely high *versus *low methylation ratios in all six of the tissue comparisons analyzed.

## Results

### Simulations predict impact of GC content and CpG distribution on fragment yield

**Figure 1:**
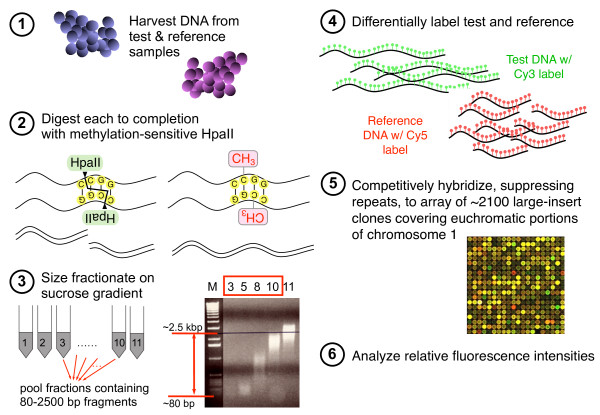
**Schematic of methylation profiling technique**. DNA is harvested from samples (**1**) and digested with methylation-sensitive enzyme, *Hpa*II, which cuts at its CCGG target site only if the CpG is not methylated (**2**). The DNA is then size-fractionated on a sucrose gradient, followed by determination of fragment size by agarose gel electrophoresis in order to choose fractions containing fragments > approximately 80 bp and < approximately 2.5 kb; in this example, fractions represented by lanes numbered 3 to 10 (**3**). Test and reference samples are then differentially labeled (**4**) and competitively hybridized to a clone-based microarray containing 2,049 clones for chromosome 1, 17 clones for the X chromosome, and 70 other clones (**5**). The signal intensities of each fluorescence channel are expressed as a ratio, log_2 _transformed, and then loess normalized on the percent GC content of clone sequences (**6**).

In our methylation-profiling technique (Figure 1), DNAs obtained from test and reference samples are digested with a methylation-sensitive restriction enzyme, *Hpa*II, which cleaves CCGG sites that are not modified by methylation. Therefore, the methylation status for each sample influences the size distribution of restriction fragments: unmethylated DNA is cut into smaller pieces than is highly methylated DNA. After digestion, fragments longer than approximately 80 bp and shorter than approximately 2.5 kb are isolated by sucrose-gradient fractionation, and test and reference DNAs are differentially labeled with fluorochromes by random priming. Thus, size fractionation is the primary determinant for distinguishing between two differentially methylated samples.

These samples are allowed to competitively hybridize to 2,136 genomic clones (bacterial artificial chromosomes (BACs), P1-derived artificial chromosomes (PACs), fosmids, cosmids) on a microarray in the presence of Cot1 DNA to suppress repetitive sequences. The array contains 2,049 clones that cover most of the euchromatic portions of human chromosome 1, 17 clones that are distributed sparsely on chromosome X, and 70 other clones. Average log_2 _ratios of test-to-reference fluorescence intensities are generated from the duplicate spots for each clone whose measurements pass quality control tests (see Methods). Since the median size of the genomic inserts of the clones on the array is 159 kb, the signal ratio for a given clone is a complex function of the methylation states of *Hpa*II sites in the sample and reference (which might both be heterogeneous mixtures of cells with different methylation patterns) and the cumulative length (minus repeats) of the resulting *Hpa*II fragments within the size range of approximately 80 to 2,500 bp in the genomic interval covered by the clone.

**Figure 2:**
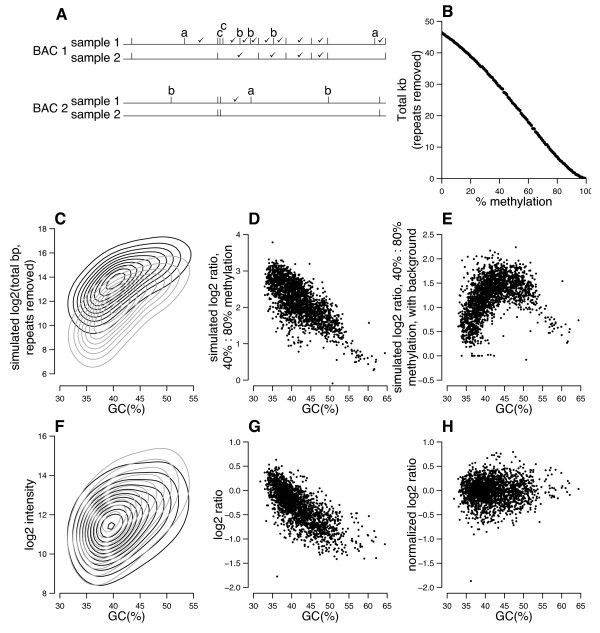
**Fragment yield simulations**. (**A**). The same methylation ratio (1:2) can give different measured ratios for BACs of different GC content. Horizontal lines represent the genomic region of a BAC in a single hybridizing sample. Sample 2 is twice as methylated as sample 1. The upper two rows represent a BAC of higher GC content than the lower rows. Vertical ticks represent unmethylated (digestable) *Hpa*II sites; check marks denote *Hpa*II fragments passing the size filter. *Hpa*II sites methylated in sample 2 but not sample 1 are marked with letters (see text). (**B**) Simulations based on BAC RP11-47A4. The amount of hybridizing material decreases with increasing methylation. (**C**) Simulation results for all array BACs. Contour plots summarize the amount of hybridizing material for all BACs at 40% methylation (black contours) and 80% (gray). (**D**) Predicted ratios when samples of 40% (test) and 80% (reference) methylation are co-hybridized. (**E**) A more complex scenario results in an opposite relationship between GC content and ratio. In this scenario, each BAC is simulated to report cross-hybridizing material in both test and reference channels equal to 5% of the number of bases of repetitive DNA it contains. (**F**) Real data for a selected array (male liver:spleen) show that, as predicted, the amount of hybridizing material is higher for higher GC content BACs (liver intensities, black contours; spleen, gray). (**G**) Measured ratios from the same array (liver:spleen) also have a strong relationship with GC content, as predicted if samples have different overall methylation levels. (**H**) GC-content-normalized ratios for the same array.

We used simulations to understand how methylation differences between samples might influence the relative fluorescence ratios measured for large-insert clones on the array. In order for the amount of hybridizing material generated from two samples to differ, the methylation state of the sample must yield, after *Hpa*II digestion, a significantly higher or lower amount of material in fragments within the size range 80 to 2,500 bp from the test sample compared with the reference. Methylation can impact the amount of product in two opposing ways (Figure 2A). For example, consider two neighboring *Hpa*II fragments, each around 2 kb in size. If the *Hpa*II site between them is methylated, then the fragments remain joined and exceed the upper size cut-off (2.5 kb). Here, methylation reduces fragment yield through the size filter (fragments adjoining sites marked "a" in Figure 2A). Conversely, methylation could increase fragment yield through the size filter for other regions, such as CpG islands, which have a high density of *Hpa*II sites (fragments adjoining sites marked "c" in Figure 2A). *Hpa*II fragments derived from unmethylated CpG islands could be excluded due to their small size (<80 bp), whereas *Hpa*II's inability to cut the same regions when methylated could generate fragments that pass the size filter. In yet other cases, methylation might have no effect on the amount of size-selected hybridizing material (sites marked "b" in Figure 2A).

In order to simulate the relative fragment yield for an entire BAC at different methylation levels, we wrote a PERL script to randomly assign each *Hpa*II site in a BAC's sequence to be methylated or unmethylated, so as to achieve various user-specified average overall levels of methylation. The script performed *in silico Hpa*II digestion of the resulting partially methylated sequence and the size-fractionation step. It also masked the sequences using RepeatMasker [56], which mimics the Cot1 repeat-suppression step necessary to achieve specificity in array hybridization. The output is total unmasked base pairs in fragments passing the size filter, which simulates labeling by random priming [57].

Our simulations show that yield of total unmasked base pairs passing the size filter from the region corresponding to any given clone on the array decreases approximately linearly with increasing average per cent methylation. Figure 2B shows the results across a range of methylation levels for BAC RP11-47A4 (AL391809), an outlying clone in the heart-*versus*-spleen comparisons and the object of experimental validation below. This negative slope indicates that the array response is dominated by methylation status at *Hpa*II sites between larger fragments where increased methylation would reduce yield through the size filter (sites of type a in Figure 2A), and that smaller *Hpa*II fragments like those in CpG islands (sites of type c in Figure 2A) have a negligible effect on the yield of size-selected fragments corresponding to any array clone. Simulations for all clones on the array show a similar negative correlation between fragment yield through the size filter and methylation level. Figure 2C and 2D display results for two selected methylation levels, demonstrating that a sample uniformly methylated at 40% of sites (Figure 2C, black contours) yields more hybridizing material for all array BACs than a sample with 80% methylation (Figure 2C, gray contours) and positive predicted log ratios for all array BACs (Figure 2D). A caveat of our simulations is that we assumed that all CpG dinucleotides had an equal chance of being methylated. More sophisticated simulations might model local heterogeneity, such as that observed in our bisulfite sequencing analyses (see Results), or the differences between CpG islands and other regions, but would need a good model to describe how methylation is distributed across sites. Additional simulations show that the relationship between fragment yield and methylation level would have a positive slope only if CpG islands comprise more than 30% of a clone's sequence (data not shown), and that is not the case for any clone on the array. Thus, signal intensity differences between clones or samples are likely to be predominated by methylation differences at CpGs outside of CpG islands.

Simulations also demonstrate a strong relationship between fragment yield and the percentage of bases that are either G or C (GC%) in the probed sequence. Figure 2A helps explain how GC content can affect measured log_2 _ratios. In this schematic example, *Hpa*II sites are relatively frequent in BAC 1, so that many fragments pass the size filter in both samples 1 and 2. The increased methylation of sample 2 causes a relatively modest reduction in hybridizing material for BAC 1. In contrast, BAC 2 has lower GC content and thus larger *Hpa*II fragments, on average, and therefore the same increased methylation level in sample 2 has a much more dramatic effect, entirely preventing any material passing the 80 to 2,500 bp size filter. This more dramatic effect would result in a more extreme measured log ratio for BAC 2 than BAC 1 despite the fact that both BACs measure the same pair of samples with the same ratio of methylation levels. Figure 2C shows the positive relationship between total base pairs in *Hpa*II fragments predicted to pass our size filter and GC% for the regions represented by the clones on our array. This positive relationship is seen at all simulated methylation levels (40% and 80% are shown in Figure 2C), except at the extreme of 100% methylation, when the DNA would not be cut at all. Moreover, the log_2 _ratio of yield for two samples simulated to have different overall methylation levels (test low, reference high) is positive for all clones on the array, but the ratios are negatively correlated with clone GC content, even though simulated methylation levels were uniform (Figure 2D). The slope of this relationship between log_2 _ratio and GC% is influenced by choice of overall methylation levels simulated for the two samples, relative simulated levels of background signals on the array spots (for example, a fixed amount of non-specific signals or signals due to incompletely suppressed repetitive elements), and allowance for variation of actual methylation levels with GC%. In fact, some combinations of these variables can yield a generally positive (but curved) slope in the relationship between log_2 _ratio and GC% (Figure 2E). To demonstrate this effect, we simulated adding cross-hybridizing signal proportional to each BAC's repeat content to both test and reference channels. The predicted ratio for BACs of low GC content is greatly reduced, because the cross-hybridizing material (equal for test and reference) outweighs the small amount of specific hybridizing material. BACs of higher GC content receive much more specific hybridizing material, so the cross-hybridizing material has less effect and the ratios are much closer to those predicted without cross-hybridization (compare Figures 2D and 2E)

Experimental data confirm a dependency on GC content. Note that we do not know the overall average levels of methylation for any of the samples we used, but any overall difference in methylation levels between the two samples being compared would result in a correlation between GC content and measured log ratio. In the example provided in Figure 2F, a comparison of liver and spleen tissue from the same donor, both test (liver) and reference (spleen) signal intensities for clones on the array increase with increasing clone GC content, and the observed log_2 _ratios decrease with clone GC content (Figure 2G). This plot shows raw ratios, but ratios normalized by intensity, a common normalization method, show a similar relationship (not shown). Because much of the variance in log_2 _ratios across the genome is accounted for by variation in GC content, we normalized log_2 _ratios for each clone based on its GC% using a loess fit of the relationship between log_2 _ratio and GC% measured for each array experiment. All subsequent plots and analyses use GC-normalized values. Note that this normalization, while necessary to correct for clone-to-clone differences in measured ratios due to differences in GC content, excludes an ability to detect any true variation in methylation levels that might correlate with GC content. The GC-normalized log_2 _ratios allow us to identify potential differences in relative methylation levels for clones with similar GC content (Figure 2H).

### Tissue differences in methylation

We next used the profiling method to detect methylation differences between tissues from the same individual. We evaluated lung, heart, liver, four regions of the brain (cerebellum, medulla oblongata, occipital lobe and pons), each from two individuals, as well as testis or ovary from single individuals. Each tissue was directly compared with spleen from the same individual to control for any inter-individual variation in genomic content (for example, segmental copy number variants and/or restriction fragment length polymorphisms). We also conducted conventional array-comparative genomic hybridization (CGH) analyses on the tissue samples to verify that no significant somatic differences in copy number existed within the same individual for sequences represented by our clone array (data not shown). The female lung sample was excluded from further analyses, because it had an unusually noisy genomic copy-number profile, suggestive of random genome instability, with relative copy number correlating strongly with methylation profile ratios (data not shown).

**Figure 3:**
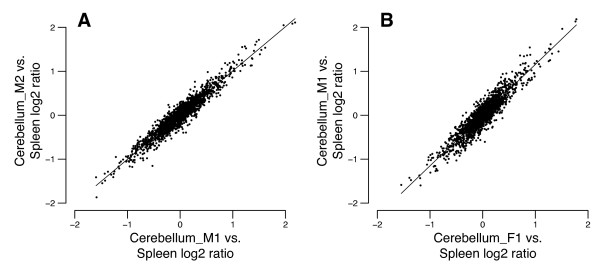
**Intra- and inter-individual reproducibility of methylation profiling method**. A very strong correlation is observed between cerebellum:spleen log_2 _ratios measured using samples from the same donor (A, Pearson *R*^2 ^= 0.92) and from different donors (B, Pearson *R*^2 ^= 0.86). See also Additional files 2, 3, 4 and 5.

To test the reproducibility of the method, we performed each tissue comparison in triplicate using tissues from each of two donors. The test replicates were taken from the same tissue DNA preparation and independently digested, size-selected, labeled, and hybridized to the array with similarly processed, but differentially labeled, spleen DNA. Replicate arrays for a given tissue comparison also usually employed different, independently processed replicates of the donor's spleen reference DNA (see Additional file 1). The log_2 _ratios for the replicates, both within and between donors, correlate very well for all tissue comparisons except those involving the male lung (Figure 3, Additional files 2, 3, 4, 5; summarized in Table 1). The poor correlation of replicate values of the three male lung-*versus*-spleen arrays (Pearson *R*^2 ^of 0.22 to 0.59) is likely because almost all of these values are close to zero, and variation is therefore predominantly experimental noise. Apart from lung, we find high Pearson correlation coefficients (*R*^2^) for within-donor replicates (median 0.88, range 0.61 to 0.95, *n *= 42) (Table 1). Thus, profiles are robustly conserved across technical replicates of both test and reference DNA. Correlation coefficients are also high when ratio values for the same tissue from different donors are compared (median 0.66, range 0.30 to 0.86, *n *= 54) (Table 1). Figure 3 shows as examples the high correlation of log_2 _ratios from the cerebellum-*versus*-spleen arrays obtained using tissues from two individuals, one male and one female (Pearson *R*^2 ^= 0.92 comparing replicates from the same donor, and *R*^2 ^= 0.86 comparing samples across donors).

**Table 1:** Pearson correlation coefficients, *R*^2^.

	***n***	**median**	**min**	**max**		***n***	**median**	**min**	**max**
**Same tissue, Same individual**					**Same tissue, Different individual**				
***All, except lung***	***42***	***0.88***	***0.61***	***0.95***	***All, except T & O***	***54***	***0.66***	***0.30***	***0.86***
cerebellum	6	0.93	0.86	0.95	cerebellum	9	0.84	0.82	0.86
pons	6	0.92	0.89	0.93	pons	9	0.67	0.64	0.71
medulla	6	0.92	0.90	0.94	medulla	9	0.66	0.62	0.68
occipital	6	0.87	0.61	0.89	occipital	9	0.66	0.52	0.70
liver	6	0.85	0.68	0.88	liver	9	0.54	0.30	0.70
heart	6	0.77	0.74	0.79	heart	9	0.51	0.39	0.56
testis	3	0.84	0.83	0.85					
ovary	3	0.90	0.87	0.91	*testis vs ovary*	*9*	*0.19*	*0.14*	*0.26*
*lung, male*	*3*	*0.31*	*0.22*	*0.59*					
									
**Different tissue, Same individual**									
All*, except lung	***108***	***0.10***	***0.00***	***0.44***	*only cerebellum represented from brain				
heart vs. ovary	9	0.40	0.38	0.44					
heart vs. testis	9	0.23	0.20	0.29					
heart vs. cerebellum	18	0.10	0.08	0.14					
heart vs. liver	18	0.06	0.01	0.19					
liver vs. ovary	9	0.02	0.00	0.07					
liver vs. testis	9	0.09	0.05	0.13					
liver vs. cerebellum	18	0.03	0.00	0.05					
cerebellum vs. ovary	9	0.11	0.09	0.14					
cerebellum vs. testis	9	0.16	0.13	0.18					
									
***All four brain parts***	***108***	***0.45***	***0.27***	***0.93***					

**Figure 4:**
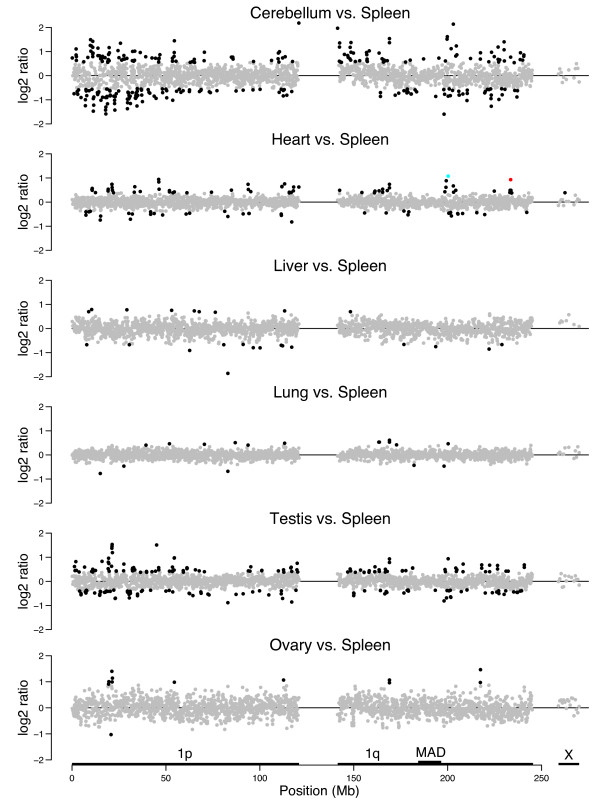
**Methylation profiles of multiple tissues show striking differences**. Cerebellum, heart, liver, lung, testis, and ovary are each profiled relative to spleen. With the exception of the ovary:spleen comparison, these representative plots were obtained using tissues from the same male donor. The log_2 _ratios for chromosome 1 clones are indicated at their relative genomic position, with the large gap representing the centromere and pericentromeric repeats in 1p11–1q21. The horizontal scale for the X chromosome is compressed; the 17 chromosome X clones on the array are actually distributed over 110 Mb. Clones highlighted in black have log_2 _ratios that deviate from the overall median, set here to 0, in either direction by >4.88 median absolute deviation (MAD) units, where MAD units are calculated using the data for the clones in the region of 1q marked 'MAD' (position 184 to 197 Mb), which showed the least biological variation within and across experiments. High log_2 _ratios are expected for regions hypomethylated relative to clones of similar GC content in the test tissue relative to spleen, whereas low log_2 _ratios are expected to represent relatively hypermethylated regions. In the heart-*versus*-spleen profile, bacterial artificial chromosome (BAC) RP11-47A4, containing part of the *RYR2 *gene, is highlighted in red and BAC RP11-90O23, containing part of *ATP2B4*, is highlighted in blue.

**Figure 5:**
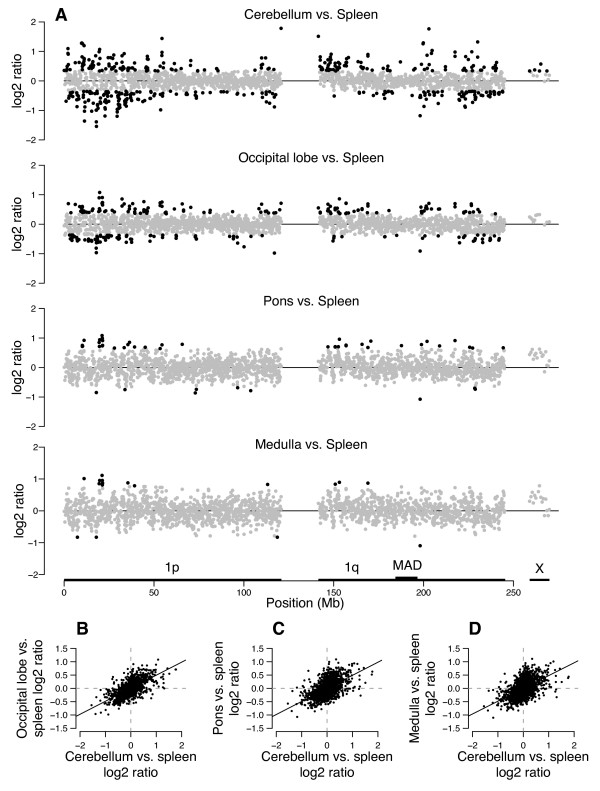
**Methylation profiles of four lobes of the brain are similar**. (**A**) Methylation profiles of four different brain regions from a female donor, each measured using the same individual's spleen as a reference. Clones highlighted in black have log_2 _ratios that deviate in either direction from the median (set at 0) by >4.88 median absolute deviation (MAD) units calculated from the region in 1q marked 'MAD'. See Figure 4 for additional details. (**B **and **C**) X-Y scatter plots illustrating the positive correlation between log_2 _ratios measured for different brain parts from female donor (each relative to her spleen). (**B**) Plot displaying a comparison of occipital lobe-*versus*-spleen and cerebellum-*versus*-spleen ratios from the female donor (F1 replicates in all cases); (**C**)Plot showing a comparison of pons-*versus*-spleen and cerebellum-*versus*-spleen ratios; (**D**)Plot showing a comparison of medulla-*versus*-spleen and cerebellum-*versus*-spleen ratios. Pearson correlation coefficients (*R*^2^) are 0.43, 0.31 and 0.29 for B, C and D, respectively, and indicate significant association at the *P *< 10^-15 ^level.

Tissues show distinctly different methylation patterns along chromosome 1 (Figures 4 and 5). In each plot, we have highlighted clones that deviate from the overall median by >4.88 median absolute deviation (MAD) units, with MAD calculated using the data for clones in a region of 1q that showed the least biological variation within and across experiments (see Methods). At one extreme, cerebellum shows a large variation in intensity ratios from region to region when compared with spleen for both donors. Regions with the greatest dynamic range in GC-normalized ratios are distributed across the chromosome, but tend to be GC- and gene-rich regions abundant in *Hpa*II sites (Additional file 6). These characteristics could potentially contribute to a greater dynamic range of measured ratios than possible in other regions of chromosome 1. At the other extreme, lung and spleen are very similar, with the profiles showing only a few sites of significant difference (Figure 4).

The methylation profiles of the various lobes of the brain map are highly correlated, with many of the peaks and valleys in the profile landscapes mapping to the same chromosomal domains (Figure 5). This similarity is best appreciated in the XY-plots provided in Additional files 2, 3, 4, their correlation coefficients summarized in Table 1, and the hierarchical cluster plots in Additional file 5. These pronounced common differences might be the cumulative result of many genes in these regions that are regulated in a tissue-specific manner, in this case brain- or spleen-specific. The median Pearson correlation coefficient of ratios measured for the four different brain portions (each compared with spleen) from the same donor is 0.45 (range 0.27 to 0.93, *n *= 108). This level of correlation approaches what we observed when comparing the same tissue taken from different individuals (see above).

In sharp contrast to the similarity of the brain tissue profiles, we observe gross differences between the methylation profiles of other tissues, perhaps reflecting regions that are differentially regulated between tissues (Figure 4, Additional file 4). The correlation coefficients are notably low when ratios measured for different organs (each relative to spleen) of the same donor are compared (median *R*^2 ^= 0.1, range 0 to 0.44 excluding lung and including a only single brain part (cerebellum) in these comparisons, *n *= 108) (Table 1). Thus, methylation profiles of the same tissue in different individuals correlate more strongly than do those of different tissues from the same individual.

The heart-*versus*-spleen methylation profiles identify an intermediate number of clones that deviate reproducibly and significantly from the median ratio in independent comparisons (Figure 4, Additional files 2 and 7). Many of these clones were not identified as outliers in other tissue comparisons, suggesting a real difference in the methylation status between heart and spleen in these genomic regions. Below, we focus our bisulfite sequencing assays of methylation state on two of these clones with outlying values.

### Bisulfite confirmation of methylation differences outside of CpG islands

We conducted bisulfite sequence analyses to confirm methylation differences between heart and spleen for sequence represented by RP11-47A4 (AL391809), which showed very high heart-*versus*-spleen log_2 _ratios in all replicates of both donors (Additional files 8 and 9). These very high relative heart:spleen ratios are predicted to reflect significantly less methylation of CpGs, and thus more frequent cutting with *Hpa*II, in heart than spleen in this locus compared with other regions of chromosome 1 after accounting for fluctuations in GC content. This clone contains part of a heart-specific gene, *RYR2*, making it an interesting candidate for confirmation of this prediction by bisulfite sequencing. These analyses also confirm our simulations, which predict that the method is most responsive to methylation differences outside of CpG islands.

**Figure 6:**
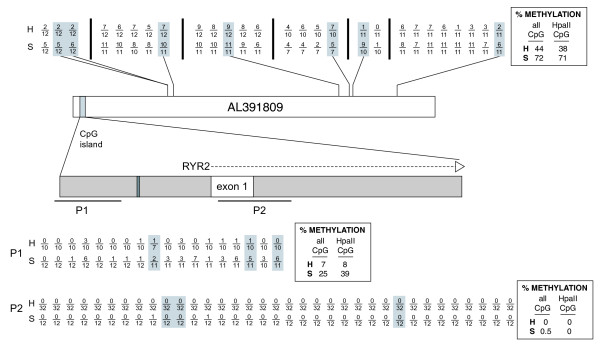
**Summary of bisulfite sequencing analyses of male heart (H) and spleen (S) DNA in the *RYR2 *gene sequence represented by BAC clone RP11-47A4 (AL391809)**. More striking methylation differences between these tissues are observed at dispersed CpGs outside of the CpG island (top) than in the island (bottom). Each CpG analyzed by bisulfite sequencing is represented by a fraction in which the denominator is the total number of sequenced clones of PCR products amplified from bisulfite-converted DNA, and the numerator is the number of times methylation was detected at that site. Data for CpGs contained within *Hpa*II sites (CCGG) are shaded gray. The results for distributed CpG sites not included in CpG islands are indicated along the top, with their relative positions in the AL391809 sequence indicated. Vertical bars separate the sites on different PCR amplicons. The CpG island is highlighted by the gray vertical bar in the schematic of the AL391809 sequence and expanded below to indicate the position of exon 1 of *RYR2 *gene (white) and the GC boxes (dark gray). Two PCR products (P1 and P2) containing multiple CpGs within this CpG island were analyzed by bisulfite sequencing, and the results are indicated at the bottom of the figure. Boxes contain overall average methylation levels for heart (H) and spleen (S) for CpGs sampled outside of the CpG island (top) and for CpGs sampled within the CpG island (bottom).

RP11-47A4 encompasses the first exon and part of the first intron of the *RYR2 *cardiac receptor gene and this gene's CpG island (Figure 6, Additional file 8); it contains no other known gene. This clone gave a high ratio only in arrays for heart *versus *spleen (>9 MAD units above the median) and cerebellum *versus *spleen (4 to 8 MAD units above median). *RYR2 *is expressed at high levels only in the heart and to a much lesser extent in some parts of the brain, albeit not cerebellum [58]; its disruption causes heart defects in humans [59-61]. We characterized the methylation state in heart and spleen of 27 CpGs distributed across this clone's sequence outside of the CpG island (Figure 6, top; Additional file 9). Seven of these CpGs are in *Hpa*II recognition sites. These *Hpa*II sites were selected for analysis from among the 174 predicted *Hpa*II sites in AL391809, because a change in their methylation status would have a large impact on yield in our procedure. The methylation state of these *Hpa*II sites would determine whether or not a fragment of 500 to 2,000 bp containing ≥ 50% unique sequence would pass the size filter (when unmethylated) or be left joined to a large flanking fragment and not contribute to the hybridization signal (when methylated). We treated DNA samples with bisulfite, amplified regions of interest by PCR using primers designed to complement bisulfite-converted sequence, and sequenced cloned amplicons from heart and spleen, respectively. Our analyses of clones of bisulfite-converted DNA show that, indeed, all but one of these seven dispersed *Hpa*II sites in AL391890 are hypomethylated in heart compared with spleen, with overall average methylation per site of 38% in heart compared with 71% in spleen (Figure 6, top; Additional file 9). Similar results were found for 20 nearby CpGs in the sequenced amplicons that are not part of *Hpa*II sites (Figure 6, top; Additional file 9)

Although our simulations predict that the differences between heart and spleen detected by the array are dominated by methylation differences such as those outside of CpG islands, we nevertheless examined the methylation state of *RYR2*'s CpG island, which contains regulatory elements, GC boxes essential for transcription of this gene, and spans a total of 181 CpGs. We successfully amplified two portions of the CpG island, containing a total of 53 CpGs. Overall, CpGs in the island were less methylated than CpGs distributed outside of the island in both heart and spleen. CpGs in the first 251-bp CpG island amplicon (P1) just upstream of the GC-boxes are approximately 3.5-fold less often methylated on average in heart than spleen, but the second 303-bp amplicon (P2) containing 35 CpGs, including five *Hpa*II sites just downstream of the GC boxes, was almost entirely unmethylated in both heart and spleen (Figure 6, bottom; Additional file 9). Only 0% and 0.5% of assayed CpGs in P2 were methylated in heart and spleen, respectively. Thus, if these combined results apply to the entire CpG island, methylation differences outside of the CpG island must account for most of the high signal ratio reported by this clone.

We also find no significant methylation differences between heart and spleen in the CpG island of *ATP2B4*, which is partially contained in RP11-90O23, another clone exhibiting a high log_2 _ratio in the heart-*versus*-spleen array comparisons (Additional file 7). *ATP2B4 *(PMCA4b) is involved in calcium homeostasis and has a role in controlling cardiac hypertrophy in response to increased load on the heart [62]. We compared the methylation status of 27 CpGs representing approximately 80% of the CpG island of *ATP2B4 *in heart and spleen using bisulfite sequence analysis. The 27 CpGs were similarly unmethylated in heart and spleen (data not shown). We found average methylation levels per site of only 0.41% and 1.0% in heart and spleen, respectively. Thus, methylation differences in the region in and around *ATP2B4 *must account for RP11-90O23's observed high log_2 _ratio in the heart-*versus*-spleen comparison, as we find no methylation differences in *ATP2B4*'s CpG island.

### Regions with outlying methylation ratios contain genes with outlying expression ratios

In order to examine the possible biological significance of the methylation ratios measured by our arrays, we compared relative methylation levels with relative expression levels of corresponding genes. In all six of the tissue comparisons examined, we found that BACs with apparent high relative levels of methylation (that is, low array ratios) tended to contain genes that were expressed at lower relative levels than BACs with lower methylation levels. This relationship is expected if regional methylation assayed by our array, like promoter methylation, is associated with suppression of transcriptional initiation [3]. In detail, we compared our results (averaged across arrays representing the same tissue comparison) with expression data obtained by Ge *et al*. [58], who hybridized RNA from each of 36 normal human tissues singly to Affymetrix oligonucleotide microarrays to determine expression levels of around 20,000 human genes (see Methods). For each gene, we calculated a ratio of expression levels for six of the tissue pairs evaluated in our methylation studies, with spleen as the reference tissue in each case (Ge *et al*. did not generate expression data for members of the remaining tissue pairs). We compared the methylation array ratios of BAC-sized genomic regions with the expression ratios of genes whose 5' ends mapped within those genomic regions, recognizing that such an analysis likely ignores facets of the complex cause-and-effect relationships between the methylation levels in different parts of a gene (promoter, body of gene, and so on) with that gene's expression levels.

**Figure 7:**
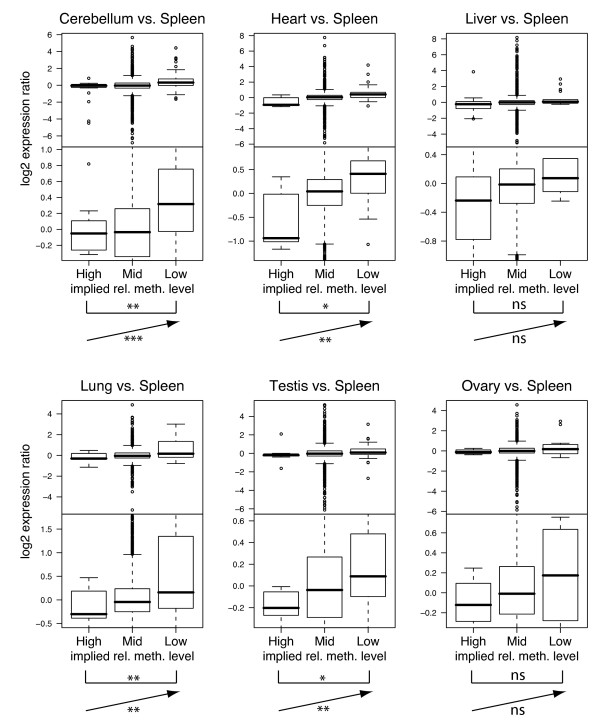
**Expression levels are higher for genes in less methylated regions**. Each pair of plots shows ratio data for a particular tissue compared with spleen, on two different y-axis scales to help illustrate expression differences. For each tissue comparison, we divided bacterial artificial chromosome (BACs) into three categories of implied relative methylation levels ('High', 'Mid' and 'Low') using median absolute deviation-based thresholds recalculated after averaging ratios across three technical replicates and two donors, if available. Note that low measured array ratios in a BAC-sized genomic region likely reflect more methylation in the test tissue than in spleen, relative to other regions of chromosome 1 with similar GC content, and are therefore labeled as 'High', and vice versa. The boxplots show the distribution of expression ratios reported by Ge *et al*. [58] for genes whose 5' ends are in BACs of each category. On each plot, the symbol within the bracket represents the *P *value obtained in a test of the null hypothesis that there is no difference in expression ratios of genes in BACs with 'Low' implied relative methylation levels versus BACs with 'High' relative methylation levels (***, 0 <*P *< 0.001; **, 0.001 <*P *< 0.01; *, 0.01 <*P *< 0.05; ns, *P *> 0.1, see Methods). The symbol next to the arrow represents the *P *value obtained in a trend test of whether expression ratios are linearly related to methylation category (coding 'High', 'Mid' and 'Low' categories as -1, 0 and 1, respectively).

While the ratios reported by our methylation-sensitive arrays and expression ratios show little overall correlation (data not shown), we noticed a relationship in the extremes of the distributions: BACs reporting lower levels of methylation in the test sample than in the reference (that is, those having very high array ratios relative to others on chromosome 1 with similar GC content) tended to contain genes whose probe sets report higher expression ratios than average, and that BACs having higher implied relative methylation levels (low methylation array ratios) tended to contain genes of lower-than-average expression ratios. In order to display and test the significance of this observation, we conservatively classified the BACs into three categories reporting 'High', 'Mid' or 'Low' implied methylation levels, using the MAD-based thresholds discussed above, and examined the expression ratios of genes mapping to each of those three categories of BACs (Figure 7). Statistical tests that account for the complex many-to-many relationships in these data (see Methods) indicate that the observed increases in relative expression level with decreased relative methylation are statistically significant in most cases (Figure 7). When less conservative criteria are used for categorizing BAC methylation ratios, statistically significant relationships are seen for all six tissues comparisons (Additional file 10); the more significant results are due in part to a greater number of BACs (and therefore genes) now in the High and Low groups.

## Discussion

DNA methylation is widely recognized as an epigenetic modification that plays a key role in specifying which portions of the genome are utilized at any given time or place in eukaryotic organisms, from plants to humans. Therefore, determining where those marks are made in the genome and how these marks are interpreted by the proteins that transcribe genes, prevent transcription of other sequences, package chromatin in the nucleus, or perform other functions on DNA, has become an important goal. We have generated chromosome-wide methylation profiles of different tissues using an approach that relies on the action of a methylation-sensitive restriction endonuclease and measurement of differences in the relative yield of size-selected fragments between two samples. Hybridization of these fragments to a tiling array of large-insert clones reports coarse regional relative differences in methylation state. Hybridization to high-resolution oligomer arrays can reveal much finer-scale fluctuations in methylation levels along the chromosome (our unpublished data).

We obtained very different methylation profiles from different organs, suggesting the existence of tissue-specific epigenetic modification patterns across chromosome 1. These patterns are conserved across individuals, as the values measured for the same tissue obtained from different donors were strongly correlated. As one might expect given the high inter-individual similarity in methylation profiles for a given tissue, replicate analyses of the same tissue from the same donor were even more strongly correlated, demonstrating that our technique gives highly reproducible results. We find that methylation profiles of the same tissue correlate better across individuals than do profiles of different tissues from the same individual. This result corroborates findings of other studies that used alternative techniques to measure relative methylation levels [31,33,63].

While most organ profiles were dissimilar, there were notable exceptions. Interestingly, the profiles for the various lobes of the brain were strongly correlated, suggesting that the shared methylation pattern of these tissues was established in a shared developmental precursor cell type and/or that the functions of these cells are sufficiently similar to be reflected in a similar pattern of epigenetic modifications. We also observe that some sites stand out as deviating from the median in multiple tissues. For example, some clones show similar relative log_2 _values in brain profiles and either testis or ovary profiles (Additional file 4). These similarities might represent spleen-specific methylation changes (spleen was used as a common reference sample) or changes common to these other organs. Other deviant sites are not shared by these different organs, such that, in fact, testis and ovary profiles are poorly correlated with each other.

Our simulations indicate that significant deviations of signal ratios from the median are influenced predominantly by methylation differences in CpG dinucleotides in *Hpa*II sites outside of CpG islands. Thus, this method complements approaches that focus on the methylation states of CpG dinucleotides in CpG islands. Our bisulfite sequencing results support the predictions of these simulations. For example, many more of the significant methylation differences that might explain the high heart:spleen signal ratio observed for the *RYR2 *locus, a gene with heart-specific function, were found outside of CpG islands than in them. An important conclusion from these results is that CpG islands and flanking DNA can have different methylation patterns, and that tissue-specific genes can be differentially methylated in regions other than their CpG islands. This conclusion is similar to that drawn from other studies which found that both the bodies and promoters of highly expressed genes are differentially methylated compared with inactive genes [30,31,38,50].

Together, our observations point to strikingly different patterns of methylation at *Hpa*II sites outside of CpG islands in various tissues. Methylation differences in the non-CpG-island compartment of the genome are largely unappreciated and merit further study to understand their possible functional consequence(s). Of all the tissues analyzed, cerebellum showed the greatest fluctuations in relative fragment yield along the chromosome, perhaps reflecting gross variation in the density of brain-expressed genes across the chromosome. In contrast, the profiles for liver, heart and lung showed much less regional variation with respect to spleen, suggesting a more even distribution (or lack) of genes expressed specifically in these tissues and associated tissue-specific epigenetic modifications.

Our findings are consistent with recent studies that have found a comparatively low frequency of tissue-specific differentially methylated regions associated with CpG-island promoters [33], while differences have been detected in CpG-poor promoters [26], outside CpG-dense promoters [36], and in CpG islands far from any known transcription start site [64]. Our data also reinforce the conclusions of Khulan *et al*. [32], who used a different approach (HELP) on mouse tissue samples to find that tissue-specific differentially methylated regions are frequent and not confined to gene promoter regions. Applying yet another methylation profiling method (RLGS), two groups found a highly significant enrichment of differentially methylated *Not*I sites away from CpG islands in mouse tissues [48,65]. Interestingly, a study by Oakes *et al*. found 8-fold more hypomethylated *Not*I sites (among 2,600 analyzed) in testis than somatic tissues, and found relatively few brain-specific differentially methylated sites [65]. In contrast, we detect many more apparently differentially methylated regions in cerebellum and other brain parts than testes when both are compared with spleen.

These observations raise the possibility that hypomethylation of CpGs outside of CpG islands might correlate with tissue-specific transcriptional activity of genes or the great many non-canonical transcripts increasingly being recognized [66]. Indeed, when we compare our measures of relative methylation of genomic clones on the chromosome 1 array and relative expression levels of constituent genes measured for the same tissue types in other individuals by Ge *et al*. [58], we find that the subsets of array clones with log_2 _values above or below our MAD-defined thresholds (implying relatively low or high methylation, respectively) tend to include genes with higher or lower relative expression levels, respectively. It is worth noting that the direction of this trend is similar to that found for promoters, not gene bodies, according to recent large-scale studies that found gene bodies to be more highly methylated in highly expressed genes than in inactive genes [31,38,50]. The regional methylation differences we observe here might correlate with the density of other marks of active/inactive chromatin as proposed by Eckhardt *et al*. [33] and/or reflect (or influence) regional compartmentalization within the nucleus [67]. Alternatively, methylation of dispersed CpGs might serve to suppress spurious transcription from cryptic promoters (as proposed for Arabidopsis [54,68]), raising the interesting question as to why the relative level of protection by methylation might vary among tissues.

In conclusion, the methylation landscapes that we have generated for various tissues suggest a complex pattern of, and possible function for, methylation differences outside of CpG islands. Future studies of regional methylation, regional transcriptional activity, and large-scale organization of the nucleus will help further our understanding of these regional epigenetic differences.

## Materials and methods

### Tissue samples

Two sets of nine tissues derived from two phenotypically normal adult individuals, one female and one male, were provided by the NCI-funded Cooperative Human Tissue Network [69] (CHTN-32364 and CHTN-32505, respectively).

### Digestion, size-fractionation, and labeling of DNA samples

We used spleen as the reference in all tissue comparisons to keep the denominator for each clone's ratio roughly constant across our experiments. Spleen DNA was used as reference for the intra-individual tissue comparisons because it was the tissue for which DNA was most abundant for both donors. Phenol- and chloroform-extracted and then isopropanol-precipitated DNA derived from test and reference tissue samples were divided into tubes (20 to 60 μg per tube) and independently processed and analyzed. Each DNA sample was digested to completion with the methylation-sensitive restriction enzyme, *Hpa*II (New England Biolabs). We monitored completion of digestion by agarose gel electrophoresis (data not shown). The digested DNA was fractionated by size on 5% to 30% sucrose gradients by using a Beckman SW-40TI swing-out rotor as previously described [53]. Fractions containing fragments smaller than 2.5 kb and greater than 80 bp as judged by gel electrophoresis were selected, pooled, and precipitated with isopropanol (Figure 1). The resulting DNA was analyzed by agarose gel electrophoresis to confirm that this process had effectively selected fragments 80 to 2,500 bp in length (data not shown). One microgram each of digested, size-selected test and reference DNA was differentially labeled with Cy3-dCTP (green) or Cy5-dCTP (red) (both GE Healthcare), respectively, by random priming using the Bioprime DNA Labeling System (Invitrogen).

### Chromosome 1 tiling array hybridization

Preparation and validation of the human chromosome 1 genomic-clone microarray was previously described [55,70]. Briefly, a total of 2,136 large-insert genomic clones were spotted in duplicate onto a glass slide. For simplicity, we will refer to this array as a 'BAC array', although the spotted clones include BACs, PACs, fosmids, and cosmids. Of these clones, 2,049 represent a tiling path covering 213 Mb, approximately 96% of the euchromatic regions of chromosome 1. Seventeen clones represent sparsely spaced segments of the X chromosome, with the first at 35 Mb and the last at 145 Mb with median spacing of approximately 6 Mb. These clones had been included on the array as controls for sex-mismatched conventional array CGH assays for other studies. The remaining 70 clones were included on the array as they were previously thought to map to chromosomes 1 or X, but are excluded from the results we report as their chromosomal mapping is now uncertain. Clone coordinates in the May 2004 assembly of the human genome sequence (Build 35) were provided by the Sanger Center and are available upon request. These coordinates typically imply clones longer than the spans shown on the NCBI or UCSC genome browsers due to trimming of sequences during genome assembly to eliminate overlapping sequence.

Labeled test and reference samples were mixed with 85 μg of Cot-1 DNA (Invitrogen) and hybridized to arrays under conditions described elsewhere [55]. Each experiment was done in triplicate using aliquots of the DNA isolated from each tissue sample, but processed independently. The replicates were independently digested, size selected, labeled and hybridized, and paired with similarly processed spleen DNA from the same donor. Replicate arrays of a given tissue usually employed different independently processed replicates of the spleen reference DNA as well (see Additional file 1).

### Array data analysis

Image acquisition was performed using an Axon 4000 B scanner and hybridization intensities were analyzed with the GenePixPro image analysis software (both Axon Instruments). For each spot, GenePixPro gave raw intensity values with the surrounding background subtracted for each wavelength scanned (635 nm and 532 nm for red and green channels, respectively). A custom R script, Normalization.r, was used to log_2_-transform these values, calculate the ratio of red to green fluorescence intensities, and apply a loess normalization procedure [71] based on GC content of the BACs (see below). A script then processed the output file to identify and eliminate clones whose duplicate spots reported significantly different normalized log_2 _ratios (that is, with standard deviation (SD) > 0.28) or had been flagged by GenePixPro as bad spots (that is, spots having less than 80% of pixels with intensities more than one SD above the background pixel intensity in either wavelength channel). We also eliminated results for subtelomeric clone RP5-857K21, as it appeared as an outlier on almost every array. The log_2 _ratios for the two spots for each remaining clone were averaged, and the median of all the clone averages was calculated and set to zero.

Our computer simulations of the method (see below and Results) indicated that relative fragment yield at a specified methylation level, as well as predicted intensity ratios for a pair of samples having different set methylation levels, vary with the GC content of the clones on the array. Therefore, we adjusted for GC content as follows: for each block of spots on each array, a loess curve was fitted to the relationship between the BACs' GC contents (in per cent) and their raw log_2 _ratios. The loess predicted value for each BAC's log_2 _ratio based on its GC content was subtracted from its real raw log ratio to give a normalized log ratio.

Using the GC-normalized data for each array, we then calculated the MAD for a contiguous set of 97 clones in 1q31–1q32 that contains relatively few *Hpa*II sites and showed little clone-to-clone variation in self-to-self comparisons and little deviation from the median in any of the sample comparisons. This group of clones was used in each array experiment as an internal measure of experimental noise to help distinguish potentially biologically meaningful deviations from experimental noise, which could vary from one array to another. This region of clones, whose midpoints are between positions 184,314,284 and 196,448,439 bp in chromosome 1's sequence in Build 35, is marked in each methylation profile with a bar marked 'MAD'. We used the MAD value as it is relatively robust to outliers and does not assume that values are normally distributed. The MAD value was calculated by subtracting the median log_2 _ratio for these selected clones from each clone's log_2 _ratio to give a deviation value; the MAD value is the median of the absolute values of these deviations. The SD of a normal distribution can be estimated to be 1.48 times the MAD value. We identified clones anywhere on chromosome 1 whose average log_2 _ratio values deviated below or above the overall median by >4.88 MAD units (that is, >3.29 times the SD), which would correspond to deviations with statistical significance at the predictive value of 0.001 based on a normal distribution with two-sided *P *value. In the self-to-self comparisons, we also used all clones to calculate the MAD and identify outliers beyond the *P *< 0.001 cutoff.

These outlying values, marked in each methylation profile, should include loci having true methylation differences between samples, since only 0.1% of the ratios drawn from a normal distribution (that is, two false-positive clones per array) are expected to deviate from the median by >4.88 MAD units. However, this threshold does not exclude all false positives due to experimental variation. The 97-clone region of 1q31–32 shows less experimental variation than the rest of the chromosome even in direct comparisons of the same tissue sample (for example, female medulla in Additional file 11). Only 0.5% of the ratios in this direct comparison deviate from the median by >4.88 MAD units when MAD is calculated over the entire array, whereas 2.2% do when MAD is calculated over the 97-clone region.

To control for potential genomic copy number differences between test and reference genomes, we also competitively hybridized 1 μg each of sonicated test and reference total DNA, mixed with 100 μg of Cot1 DNA (Invitrogen), to replicas of the same microarrays using the same conditions as described above [55]. No significant deviations in copy number were observed across chromosome 1 in any tissue sample relative to the spleen sample from the same donor in these conventional array-based comparative genomic hybridization (array CGH) assays, except the female lung sample (data not shown). Because log_2 _ratios for the methylation-profiling array correlated very strongly with the wildly varying log_2 _ratios from the conventional CGH array for this sample (in isolation, each profile appeared to have large random noise), we eliminated this sample from further analysis. Genomic content abnormalities were noted for this lung sample in another study [72].

Note that it is not possible using this comparative method alone to determine what ratio represents an equivalent methylation state in test and reference DNA, as is possible with some sequencing-based methods (for example, [36,49]). Thus, while a normalized log_2 _ratio of 0 (the median) might represent equivalent actual levels of methylation in a comparison of two normal tissue samples, it probably would not in a comparison of cells deficient in DNA methyltransferase activity and control cells.

### Methylation simulation

In order to understand the response of our array to differently methylated genomes, we developed a PERL script, METHBATCHSIM, to simulate methylation states for a sequence of interest, such as a particular clone on the array, and the corresponding yield of fragments through our methylation-profiling procedure. First, we generated a list of *Hpa*II restriction sites on the sequence and used RepeatMasker [56] to generate a repeat-masked sequence file. METHBATCHSIM reads in the *Hpa*II and masked-sequence files and randomly designates which restriction sites in each clone contain methylated CpGs and which are unmethylated, for a specified overall methylation proportion. Specifically, we used the rbinom function of the R package [73] to randomly assign methylation status of each site, using the desired overall methylation fraction (between 0% and 100%, in steps of 1%) as the 'prob' parameter, the number of restriction sites in the BAC as the 'n' parameter, and the 'size' parameter set at 1. The sequence is then virtually digested with *Hpa*II based on the assigned methylation states, and we determine which of the resulting fragments are within the specified size range (80 to 2,500 bp). We simulated labeling by random priming by summing the total unmasked base pairs in all fragments meeting the size-range criterion. The final output summarizes these totals, averaged over 1,000 simulation runs. We repeated this simulation using the sequence of each clone on the array.

### Other bioinformatic analyses

CpG island coordinates were taken from the UCSC Genome Browser [74] CpG Island track, where CpG islands are defined as regions of >200 bp with GC content ≥ 50% and a ratio >0.6 of observed number of CG dinucleotides to expected number on the basis of the numbers of Gs and Cs in the segment. Gene coordinates were obtained from the UCSC's RefSeq track.

### Expression analysis

In order to test whether the relative methylation levels we measured relate to transcription levels, we compared our results with expression data obtained by Ge *et al*. [58], who hybridized RNA from each of 36 normal human tissues singly to Affymetrix oligonucleotide microarrays to determine expression levels of approximately 20,000 human genes. No RNA was available to study expression levels in samples from the same donors used for our methylation studies, but because methylation array ratios were highly correlated between the two donors we studied, it seemed reasonable to assume that methylation states would be similar in the tissues of donors used by Ge *et al*. and, therefore, reasonable to think that if any consistent correlation between regional methylation and expression levels exists, it might be observed even if different tissue donors are used for the methylation and expression arrays. We averaged methylation array ratios for the same tissue comparisons (that is, across sets of six arrays for each of cerebellum, heart and liver, and across sets of three arrays for each of lung, testis and ovary, compared with spleen in all cases). We used these cross-array averages to recalculate the MAD-based thresholds used to classify BACs as having outlying methylation array values.

Starting with Ge *et al*.'s raw probe-level expression-array data (GEO series GSE2361), we applied standard background adjustment, normalization and log-transformation methods using the robust multi-array average algorithm from Bioconductor's affy package [75] to obtain expression levels for each probe set (a probe set is a set of around 20 oligonucleotide probes that together represent a portion of a single gene). For each of the six tissue comparisons made (Figure 7, Additional file 10), we combined normalized expression results for the relevant tissue types to obtain log_2 _expression ratios. A table of Affymetrix probe set names, their corresponding gene symbols, and genomic coordinates of those genes in Build 35 of the human genome assembly was obtained from the SCGAP Hematopoietic Stem Cells Project [76]. We then plotted the expression ratios of probe sets corresponding to genes whose 5' ends mapped in BACs of each three implied categories of implied relative methylation levels: 'High', 'Mid' or 'Low'. These conservative classifications correspond to BACs with ratios on the methylation-sensitive arrays that exceed MAD-based thresholds discussed above (that is, for Figure 7, 4.88 MAD units from the median, a threshold chosen such that we would expect approximately 0.05% of BACs on the array to fall in each of the Low or High categories by chance). In each boxplot, the median is indicated by the thick line, the box spans the middle two quartiles of the distribution, the small circles are outliers, and the whiskers extend to the most extreme data point which is no more than 1.5 times the length of the box away from the box.

In order to test the statistical significance of the observed differences in relative expression ratios between genes in BACs of different methylation ratio categories, we needed to account for the many-to-many relationships that exist between BACs, genes and probe sets. A BAC can contain more than one gene, a gene's 5' end can be present in more than one overlapping BAC, and some genes are represented on the expression array by more than one probe set (located in different parts of the gene). We therefore fitted generalized estimating equations to the data (using R's geeglm function from the geepack package [77]), treating datapoints from the same BAC as groups. We also gave each BAC-probe set datapoint a weight equal to the reciprocal of the number of times its gene symbol appeared in the table of all BAC-probe set pairs (that is, a gene represented by three probe sets and whose 5' end is in two overlapping BACs would receive a weight of 1/6 for each of its six datapoints). For each tissue comparison, we performed two statistical tests: (a) a test of whether expression ratios differ between BACs in the 'High' and 'Low' methylation categories (*P *values on square brackets, Figure 7 and Additional file 10); (b) a trend test for whether expression ratios are linearly related to methylation category, when methylation levels are coded as -1 (for low array ratio and 'High' implied relative methylation level), 0 ('Mid'), or 1 (for high array ratio and 'Low' implied relative methylation level) (*P *values on arrows, Figure 7 and Additional file 10). In each test, we used the 'Gaussian' family to model variation in expression ratios and 'independence' as the correlation structure.

### PCR, cloning and sequencing of bisulfite-modified DNA

Total genomic DNA was extracted from male heart and spleen samples, and unmethylated cytosines were converted to uracil by bisulfite treatment according to published protocols [78]. Briefly, each DNA sample was first digested with *Bam*H1, the enzyme was removed by phenol and chloroform extraction, and the DNA was subsequently ethanol precipitated and resuspended in H_2_O. NaOH was added to each DNA sample to a final concentration of 0.3 M, and the DNA was denatured for 2 min at 97°C followed by incubation for 30 min at 39°C. A solution of sodium bisulfite and hydroquinone was immediately added to 3.3 M and 0.67 mM final concentrations, respectively, and samples were incubated for 16 h at 55°C with 95°C spikes for 5 min every 3 h. Samples were desalted using QIAquick PCR Purification columns (Qiagen) according to the manufacturer's instructions. NaOH was added to each converted sample to a final concentration of 0.3 M, and the samples were incubated at 37°C for 15 min to remove excess sodium bisulfite. DNA samples were then ethanol precipitated and resuspended in 100 μl H_2_O.

Primers for selected regions were designed using the BiSearch Web Server [79] and are provided upon request. Each amplification was done with touchdown PCR using 2 μl Ex-Taq enzyme (Takara Mirus Bio) and 0.5 μl of 50 μM primers in a total volume of 25 μl with the following conditions: 95°C for 2 min, 30 cycles of 95°C for 30s, 66°C for 30s (stepping down 2°C every 2 cycles until 56°C), and 72°C for 1 min, followed by 72°C for 10 min.

Amplified products from converted DNA were gel-purified with QIAquick Gel Extraction columns (Qiagen) according to the manufacturer's instructions and subsequently cloned into the pCR2.1 plasmid vector using the TOPO TA Cloning Kit (Invitrogen). Clones with inserts were identified by PCR amplification using M13 reverse and T7 forward primers using TOPO cloning instructions. Amplified M13-T7 products were purified with Sephacryl S-300 (Amersham BioSciences). Seven to thirty-two clones were then sequenced successfully from each PCR product using the same primers on an ABI 3730 (Applied Biosystems). Sequences were aligned and analyzed using PhredPhrap and Consed [80-82].

All array data relevant to this manuscript have been submitted to the Gene Expression Omnibus (GEO) under accession number GSE12925.

## Abbreviations

*ATP2B4*: Ca-transporting plasma membrane ATPase isoform 4; BAC: bacterial artificial chromosome; CGH: comparative genomic hybridization; MAD: median absolute deviation; PAC: P1-derived artificial chromosome; *RYR2*, ryanodine cardiac receptor 2; SD: standard deviation.

## Competing interests

The authors declare that they have no competing interests.

## Authors' contributions

CDB, ER, and RKT carried out the methylation profiling experiments. CDB, ER, JMY, UM and BJT analyzed the array data. ER carried out the bisulfite sequencing, and ER and JMY analyzed the data. CFL was responsible for BAC array design and fabrication. EEE contributed tissue samples. LH participated in the statistical analysis. JMY performed simulation studies. JPD, SH, EEE, and BJT participated in the overall design and coordination of the study. CDB, ER, JMY, and BJT wrote the manuscript. All authors read and approved the final manuscript.

## Supplementary Material

Additional File 1 **Table S1**. Replicates of test and spleen reference paired for each array. Click here for file

Additional File 2 **Figure S1**. Scatter plots are displayed above the diagonal to illustrate pairwise comparisons between results of different methylation profiling microarray experiments. The corresponding Pearson *R*^2 ^correlation coefficients are shown below the diagonal. This figure includes three replicate arrays each for various tissue samples from each of two donors. Female lung is excluded due to observed genomic copy number deviations. Only cerebellum is included in this set as a representative brain part due to space considerations. The reference tissue in each array is spleen, from the same donor as the test tissue. The red dashed line in each plot indicates the linear regression fit. Cells along the diagonal provide tissue and replicate information. Abbreviation: Cere, Cerebellum. Replicates are indicated by the suffix following the underscore: M1, M2, and M3 are replicates from the male donor; F1, F2, F3 are replicates from the female donor. Most replicate arrays for a given tissue employed different replicates of the spleen reference (see Additional file 1). Click here for file

Additional File 3 **Figure S2**. Scatter plots are displayed above the diagonal to illustrate pairwise comparisons between results of different methylation profiling microarray experiments performed using various brain tissue samples. The corresponding Pearson *R*^2 ^correlation coefficients are shown below the diagonal. This figure includes three replicate arrays for each tissue sample from each of two donors. Abbreviations: Cere, Cerebellum; Occi, Occipital Lobe; Med, Medulla. See Additional file 2 legend for additional details. Plots comparing F1 brain samples are also shown in Figures 5B, 5C, and 5D. Click here for file

Additional File 4 **Figure S3**. Scatter plots are displayed above the diagonal to illustrate pairwise comparisons between results of different methylation profiling microarray experiments performed using various tissues from two donors. Here one representative replicate of each tissue from each donor is included in the pairwise comparison dataset. The corresponding Pearson *R*^2 ^correlation coefficients are shown below the diagonal. See Additional file 2 legend for additional details. Click here for file

Additional File 5 **Figure S4**. Hierarchical clustering of methylation profiles from 45 fractionated DNA samples from 15 tissue samples. GC-normalized ratios were clustered using Manhattan distances and the Ward method of hierarchical clustering using R's hclust function. The dendrogram demonstrates that there is tight correlation within each set of triplicate samples and between samples from the same tissue in different individuals. Abbreviations: Med, Medulla; Cere, Cerebellum; Occi, Occipital Lobe. Click here for file

Additional File 6 **Figure S5**. Chromosome 1 G-banding, GC%, gene density, and *Hpa*II site distribution. The G-banding pattern of chromosome 1 is aligned to plots of (1) of the percentage of G or C nucleotides in each array clone as a function of the clone's position along the chromosome, (2) of the number of start sites of RefSeq genes in non-overlapping windows of 1 Mbp along the chromosome (obtained from the UCSC Genome Browser RefSeq track), and (3) the number of possible *Hpa*II fragments in the 80 to 2,500 bp size range in each array clone as a function of the clone's position along the chromosome. The thick black bar indicates the region of 97 clones used to calculate median absolute deviation values in all experiments. Click here for file

Additional File 7 **Figure S6**. Reproducibility of methylation profiles across three experiments in which heart DNA is compared with spleen using tissue from the male donor. DNA was extracted from a single heart and single spleen tissue sample, and each DNA preparation was then divided into aliquots that were independently processed through *Hpa*II digestion, size selection, labeling, and array hybridization. Replicates are identified as M1, M2, and M3 for heart, and M1, M6, and M7 for spleen, where M denotes the male donor. (A) Profiles of log_2 _ratios as function of genomic position. Clones highlighted in black, red, or blue have log_2 _ratios that deviate from the median by >4.88 median absolute deviation, which was calculated over the region indicated with a wide bar. The clone whose value is indicated in red is RP11-47A4, which contains part of the *RYR2 *gene; the value in blue is for clone RP11-90O23, which contains *ATP2B4*. Sequences encompassed by these clones are the subject of our bisulfite sequencing analyses. (B) Correlations of log_2 _ratios measured in the three pairs of experiments. Scatterplots are shown above the diagonal; values below the diagonal are the corresponding Pearson correlation coefficients (*R*^2^). Click here for file

Additional File 8 **Figure S7**. Enlarged view of clones on the array around the region containing the ryanodine cardiac receptor 2 gene (*RYR2*). Relative positions of bacterial artificial chromosomes (BACs) covering the region flanking RP11-47A4 (AL391809) and their corresponding mean log_2 _ratios (dotted lines are at ± 1 standard error of the mean) in six heart-*versus*-spleen methylation profiles (three using DNA prepared from tissue from the female donor, and three utilizing DNA from tissue from the male donor). RP11-47A4 has the highest mean log_2 _ratio value of the five BACs shown here. The relative positions of *RYR2 *exons are indicated by black dots (size not to scale) and the *RYR2 *CpG island, which coincides with *RYR2*'s first exon is indicated. *RYR2 *extends approximately 340 kb beyond the region shown. Click here for file

Additional File 9 **Figure S8**. Methylation status of CpGs in RP11-47A4 (AL391809), which contains the *RYR2 *gene, as determined by sequencing of PCR products amplified after bisulfite conversion of unmethylated cytosines to uracil. These data are summarized in Figure 6. Here, the raw data are provided for each sequenced amplicon from heart or spleen. Filled squares represent methylated CpG dinucleotides, open squares unmethylated CpGs, and gray background shading denotes CpGs that are part of *Hpa*II restriction sites. Click here for file

Additional File 10 **Figure S9**. Expression ratios tend to be higher for genes whose 5' ends fall in regions of lower methylation (that is, in bacterial artificial chromosomes (BACs) with higher methylation array ratios) than those in regions of higher methylation. Data are presented as in Figure 7, but here BACs are classified in the 'High' and 'Low' categories using a less conservative methylation array ratio threshold. This less conservative threshold is defined as 2.91 * median absolute deviation (MAD) units below and above the median value. At the 2.91 * MAD threshold, we expect approximately 2.5% of BACs on the array to fall into each of the 'High' and 'Low' categories purely by chance (that is, an overall false-positive rate of 5% of array BACs). Figure 7 conservatively uses 4.88 * MAD, such that the false-positive rate is expected to be only around 0.1% of the BACs on the array. The increased number of BACs in the 'High' and 'Low' categories using this less conservative approach likely gives the statistical tests greater power, explaining the greater significance of most *P *values shown here as compared with Figure 7. Click here for file

Additional File 11 **Figure S10**. Methylation profile in which two independently processed aliquots of DNA isolated from medulla tissue from the same female donor are directly compared. The log_2 _ratios for chromosome 1 clones are indicated at their relative genomic position, with the large gap representing the centromere and pericentromeric repeats in 1p11–1q21. The horizontal scale for the X chromosome is compressed; the 17 chromosome X clones on the array are distributed over 110 Mb. Clones highlighted in black or red have log_2 _ratios that deviate from the overall median, set here to 0, in either direction by >4.88 median absolute deviation (MAD) units, where MAD units are calculated using the data for the clones in the region of 1q marked 'MAD', which showed the least biological variation within and across experiments. Clones highlighted in red deviate from the overall median by ± 4.88 MAD, calculated using all values. Click here for file
