# Supplementary figures and images for: Tissue-specific variation in DNA methylation levels along human chromosome 1
Source: Epigenetics Chromatin. 2009 Jun 8;2:7. doi: 10.1186/1756-8935-2-7 (PMC2706828; doi:10.1186/1756-8935-2-7)

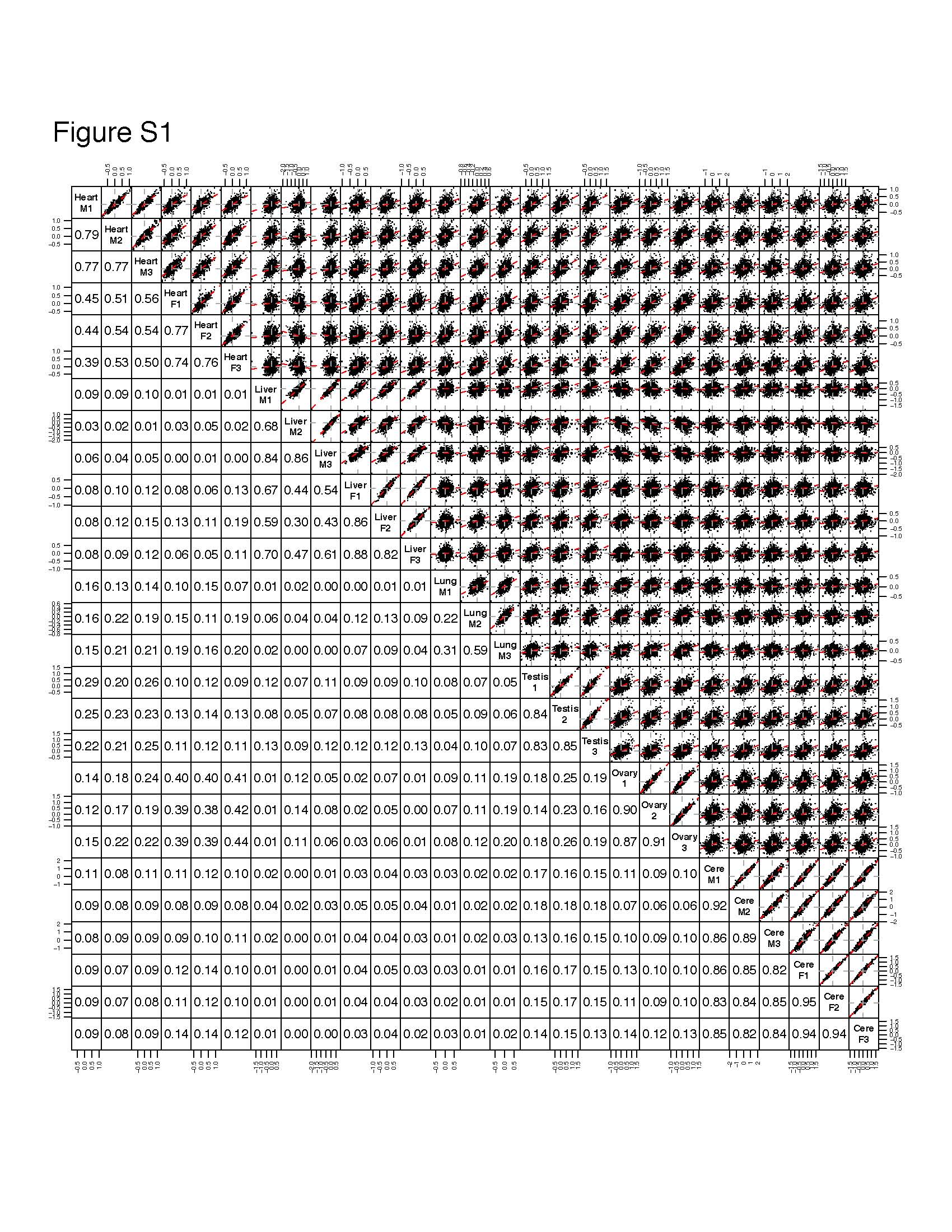

Supplement: Additional File 2 — Figure S1. Scatter plots are displayed above the diagonal to illustrate pairwise comparisons between results of different methylation profiling microarray experiments. The corresponding Pearson R2 correlation coefficients are shown below the diagonal. This figure includes three replicate arrays each for various tissue samples from each of two donors. Female lung is excluded due to observed genomic copy number deviations. Only cerebellum is included in this set as a representative brain part due to space considerations. The reference tissue in each array is spleen, from the same donor as the test tissue. The red dashed line in each plot indicates the linear regression fit. Cells along the diagonal provide tissue and replicate information. Abbreviation: Cere, Cerebellum. Replicates are indicated by the suffix following the underscore: M1, M2, and M3 are replicates from the male donor; F1, F2, F3 are replicates from the female donor. Most replicate arrays for a given tissue employed different replicates of the spleen reference (see Additional file 1). [file 1756-8935-2-7-S2.jpeg]

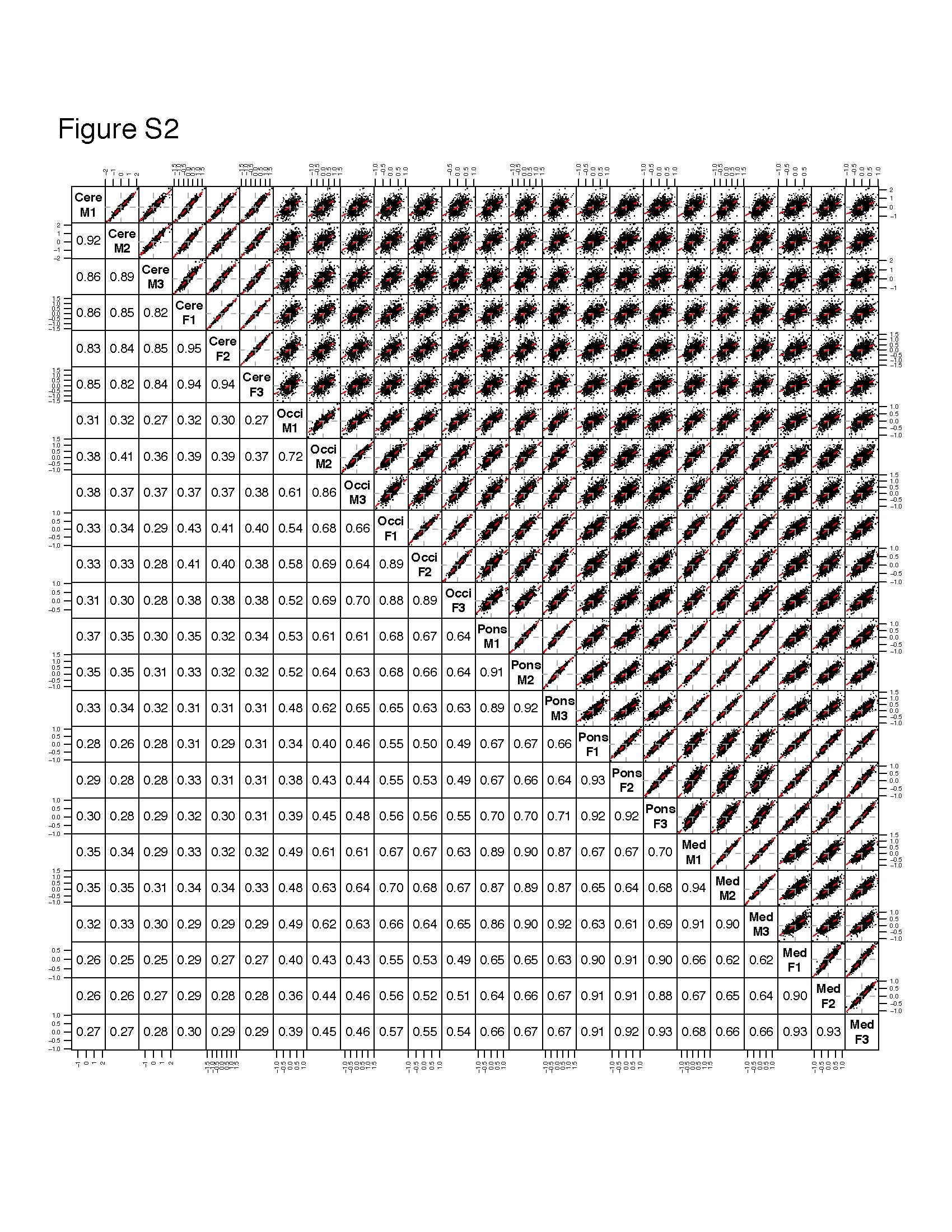

Supplement: Additional File 3 — Figure S2. Scatter plots are displayed above the diagonal to illustrate pairwise comparisons between results of different methylation profiling microarray experiments performed using various brain tissue samples. The corresponding Pearson R2 correlation coefficients are shown below the diagonal. This figure includes three replicate arrays for each tissue sample from each of two donors. Abbreviations: Cere, Cerebellum; Occi, Occipital Lobe; Med, Medulla. See Additional file 2 legend for additional details. Plots comparing F1 brain samples are also shown in Figures 5B, 5C, and 5D. [file 1756-8935-2-7-S3.jpeg]

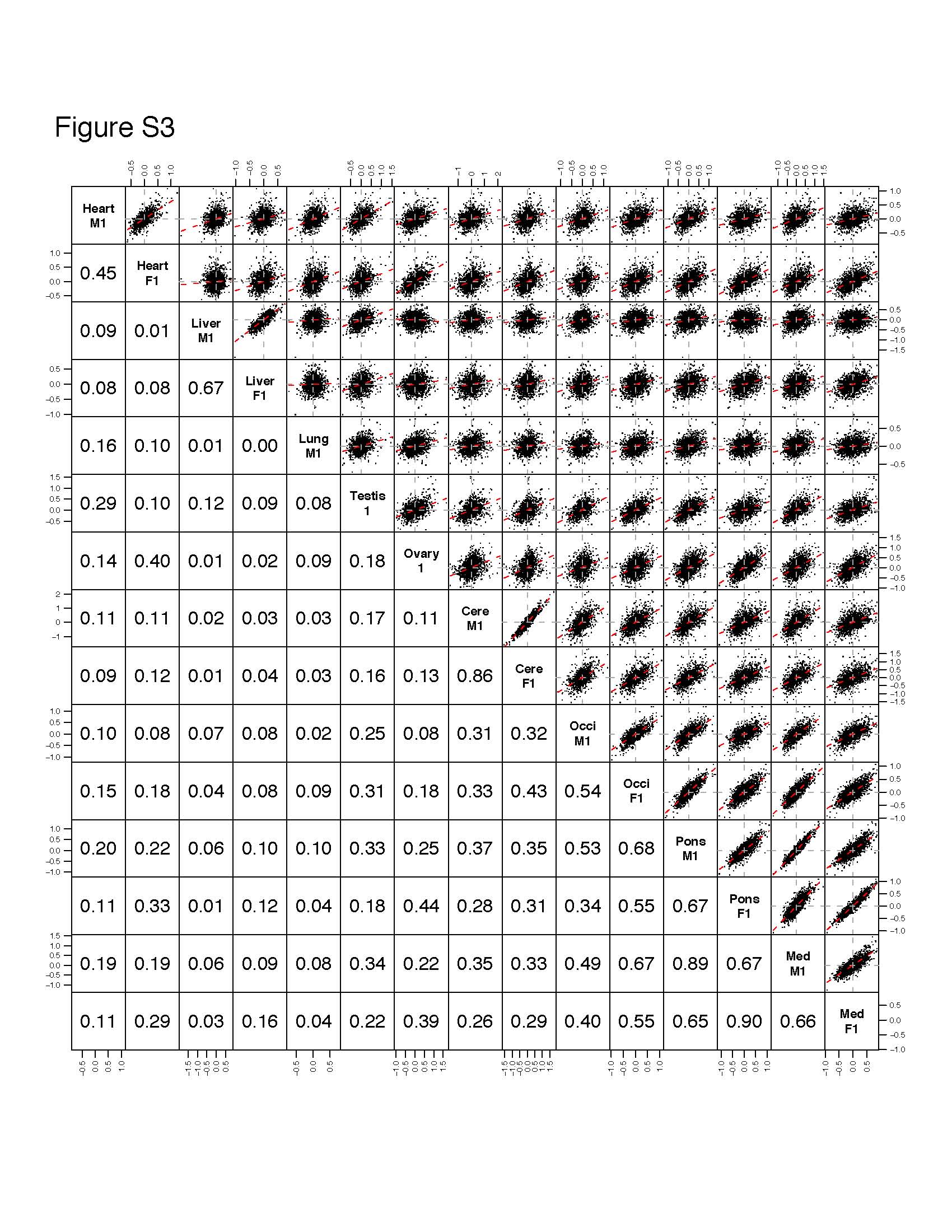

Supplement: Additional File 4 — Figure S3. Scatter plots are displayed above the diagonal to illustrate pairwise comparisons between results of different methylation profiling microarray experiments performed using various tissues from two donors. Here one representative replicate of each tissue from each donor is included in the pairwise comparison dataset. The corresponding Pearson R2 correlation coefficients are shown below the diagonal. See Additional file 2 legend for additional details. [file 1756-8935-2-7-S4.jpeg]

Figure S4

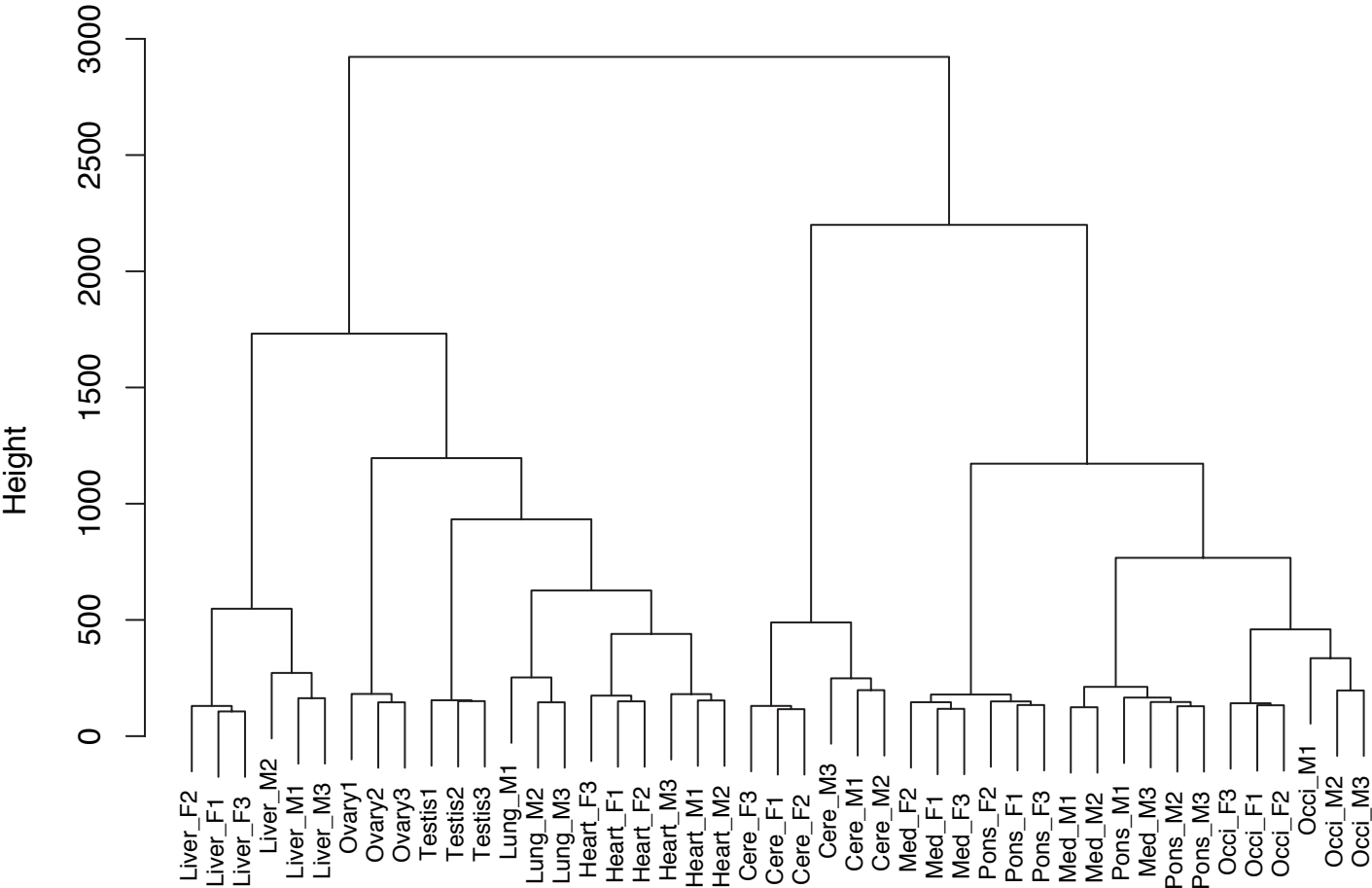

Supplement: Additional File 5 — Figure S4. Hierarchical clustering of methylation profiles from 45 fractionated DNA samples from 15 tissue samples. GC-normalized ratios were clustered using Manhattan distances and the Ward method of hierarchical clustering using R's hclust function. The dendrogram demonstrates that there is tight correlation within each set of triplicate samples and between samples from the same tissue in different individuals. Abbreviations: Med, Medulla; Cere, Cerebellum; Occi, Occipital Lobe. [file 1756-8935-2-7-S5.pdf]

Figure S5

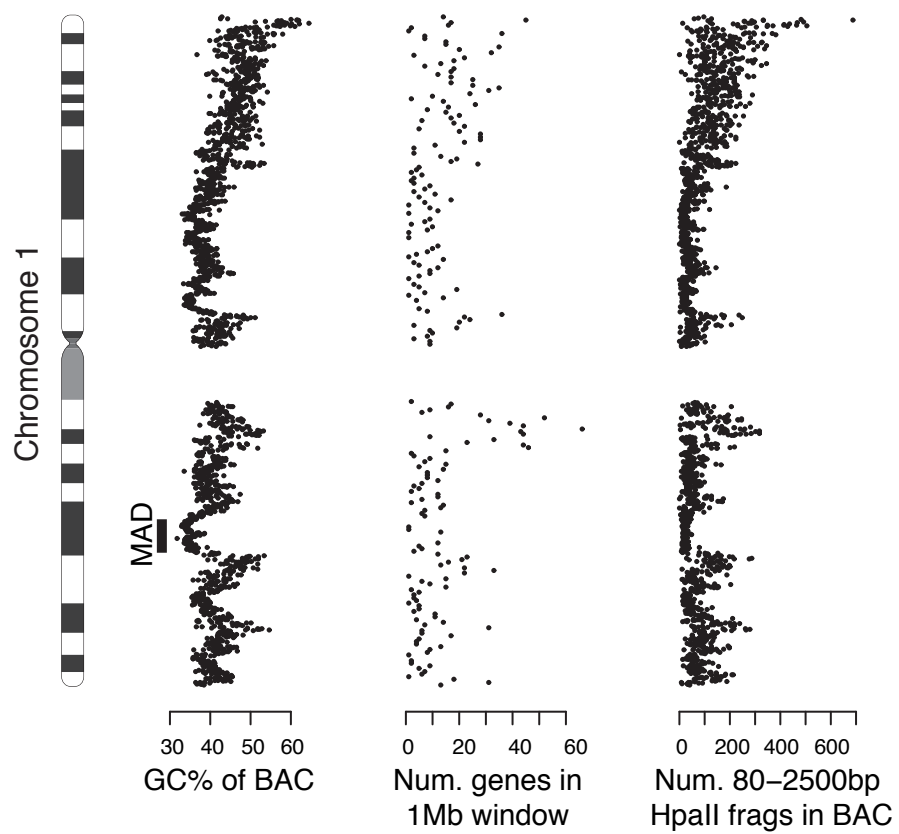

Supplement: Additional File 6 — Figure S5. Chromosome 1 G-banding, GC%, gene density, and HpaII site distribution. The G-banding pattern of chromosome 1 is aligned to plots of (1) of the percentage of G or C nucleotides in each array clone as a function of the clone's position along the chromosome, (2) of the number of start sites of RefSeq genes in non-overlapping windows of 1 Mbp along the chromosome (obtained from the UCSC Genome Browser RefSeq track), and (3) the number of possible HpaII fragments in the 80 to 2,500 bp size range in each array clone as a function of the clone's position along the chromosome. The thick black bar indicates the region of 97 clones used to calculate median absolute deviation values in all experiments. [file 1756-8935-2-7-S6.pdf]

Figure S6

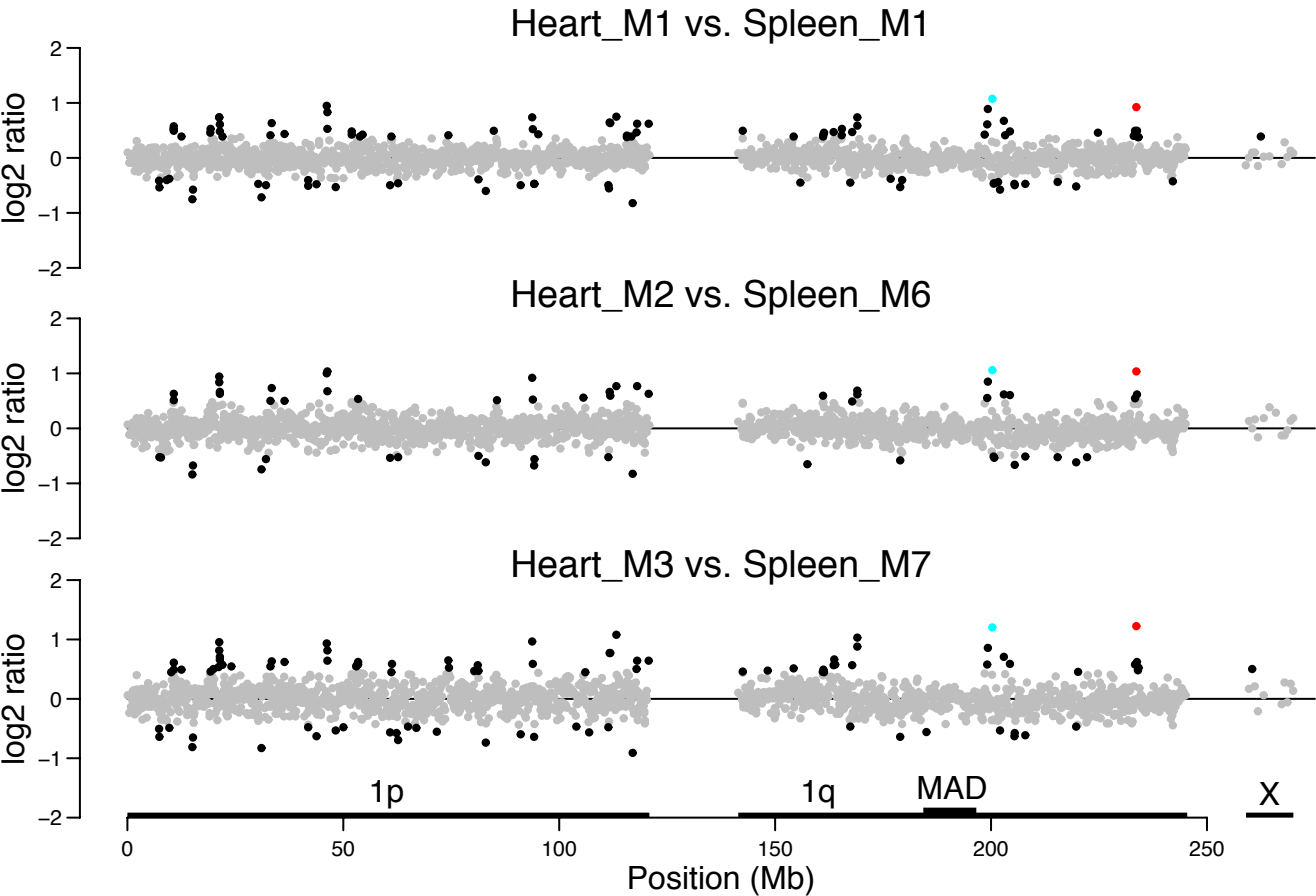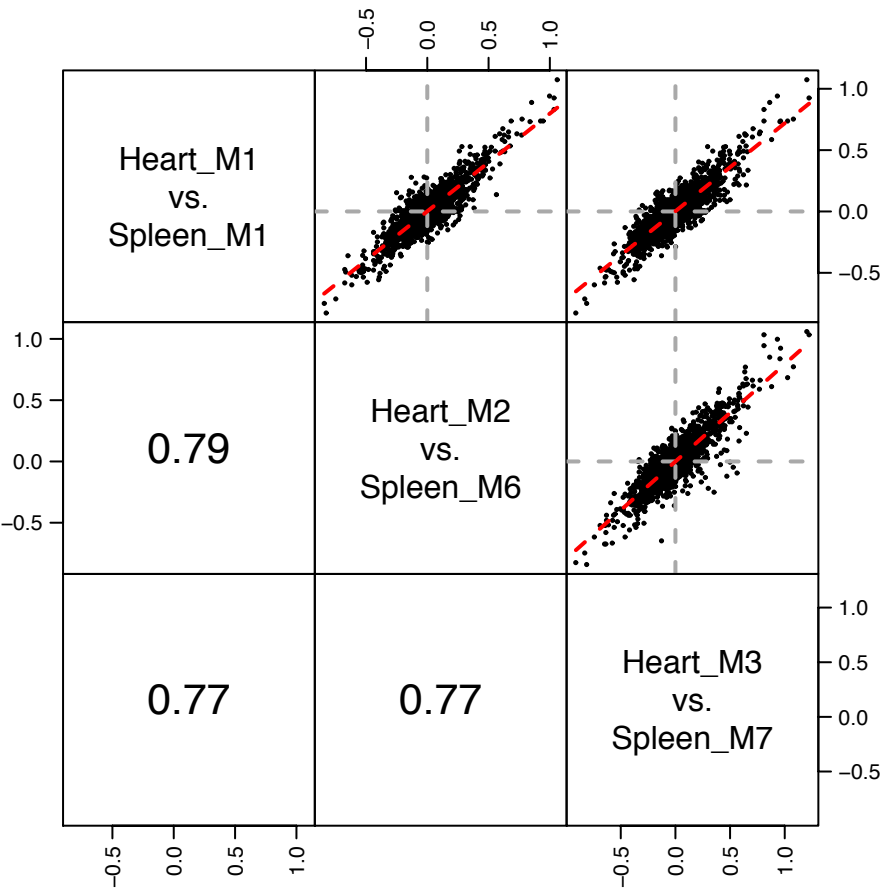

Supplement: Additional File 7 — Figure S6. Reproducibility of methylation profiles across three experiments in which heart DNA is compared with spleen using tissue from the male donor. DNA was extracted from a single heart and single spleen tissue sample, and each DNA preparation was then divided into aliquots that were independently processed through HpaII digestion, size selection, labeling, and array hybridization. Replicates are identified as M1, M2, and M3 for heart, and M1, M6, and M7 for spleen, where M denotes the male donor. (A) Profiles of log2 ratios as function of genomic position. Clones highlighted in black, red, or blue have log2 ratios that deviate from the median by >4.88 median absolute deviation, which was calculated over the region indicated with a wide bar. The clone whose value is indicated in red is RP11-47A4, which contains part of the RYR2 gene; the value in blue is for clone RP11-90O23, which contains ATP2B4. Sequences encompassed by these clones are the subject of our bisulfite sequencing analyses. (B) Correlations of log2 ratios measured in the three pairs of experiments. Scatterplots are shown above the diagonal; values below the diagonal are the corresponding Pearson correlation coefficients (R2). [file 1756-8935-2-7-S7.pdf]

Figure S7

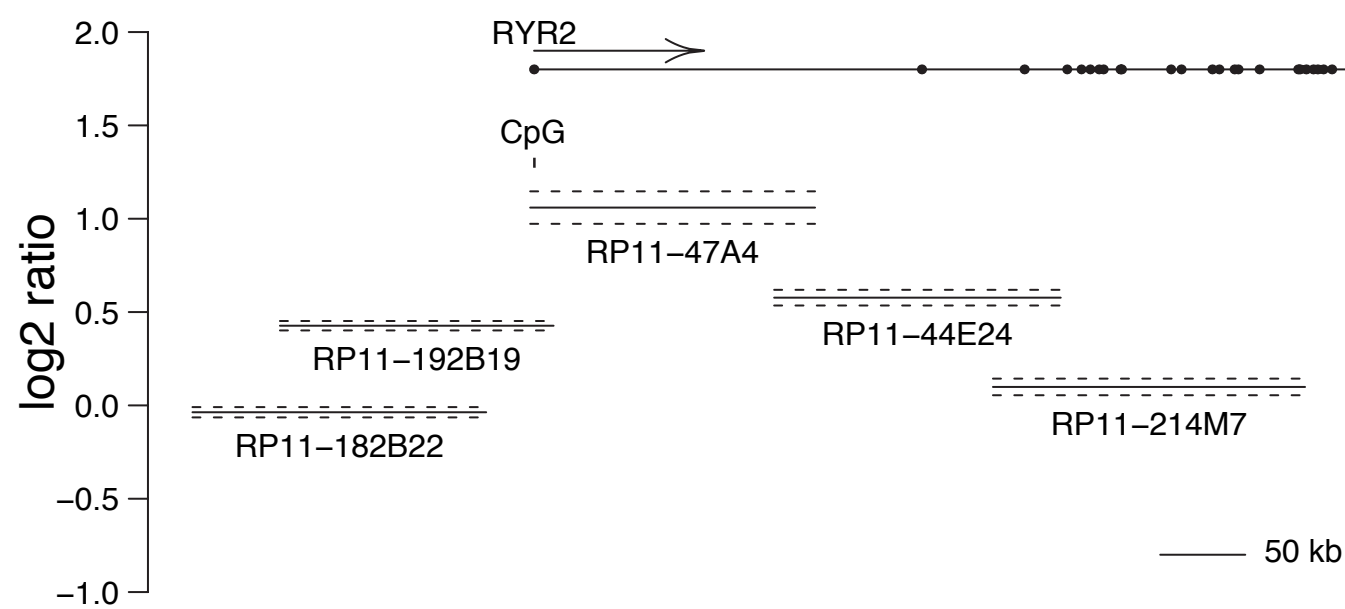

Supplement: Additional File 8 — Figure S7. Enlarged view of clones on the array around the region containing the ryanodine cardiac receptor 2 gene (RYR2). Relative positions of bacterial artificial chromosomes (BACs) covering the region flanking RP11-47A4 (AL391809) and their corresponding mean log2 ratios (dotted lines are at ± 1 standard error of the mean) in six heart-versus-spleen methylation profiles (three using DNA prepared from tissue from the female donor, and three utilizing DNA from tissue from the male donor). RP11-47A4 has the highest mean log2 ratio value of the five BACs shown here. The relative positions of RYR2 exons are indicated by black dots (size not to scale) and the RYR2 CpG island, which coincides with RYR2's first exon is indicated. RYR2 extends approximately 340 kb beyond the region shown. [file 1756-8935-2-7-S8.pdf]

Figure S8

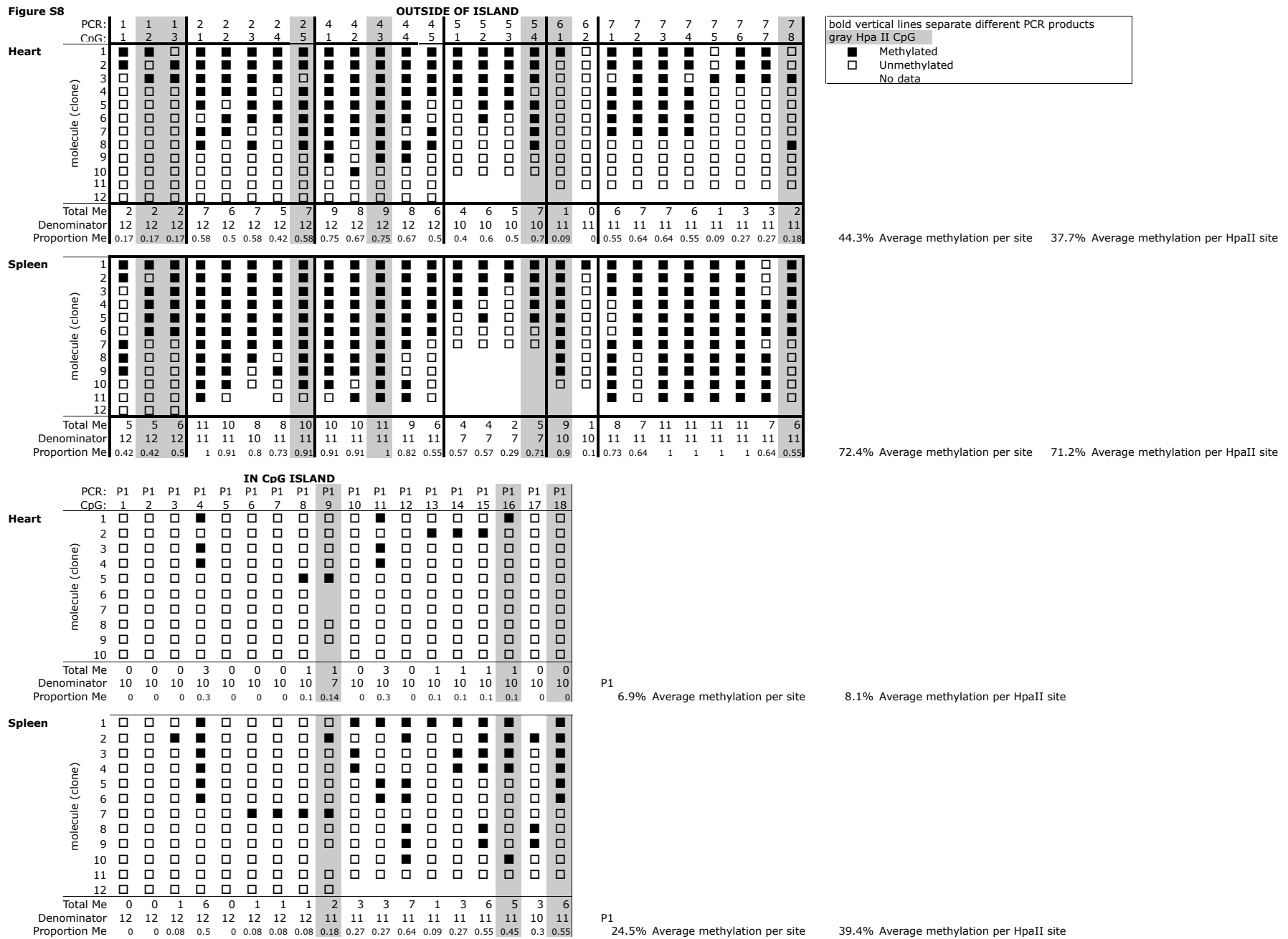

Supplement: Additional File 9 — Figure S8. Methylation status of CpGs in RP11-47A4 (AL391809), which contains the RYR2 gene, as determined by sequencing of PCR products amplified after bisulfite conversion of unmethylated cytosines to uracil. These data are summarized in Figure 6. Here, the raw data are provided for each sequenced amplicon from heart or spleen. Filled squares represent methylated CpG dinucleotides, open squares unmethylated CpGs, and gray background shading denotes CpGs that are part of HpaII restriction sites. [file 1756-8935-2-7-S9.pdf]

Figure S9

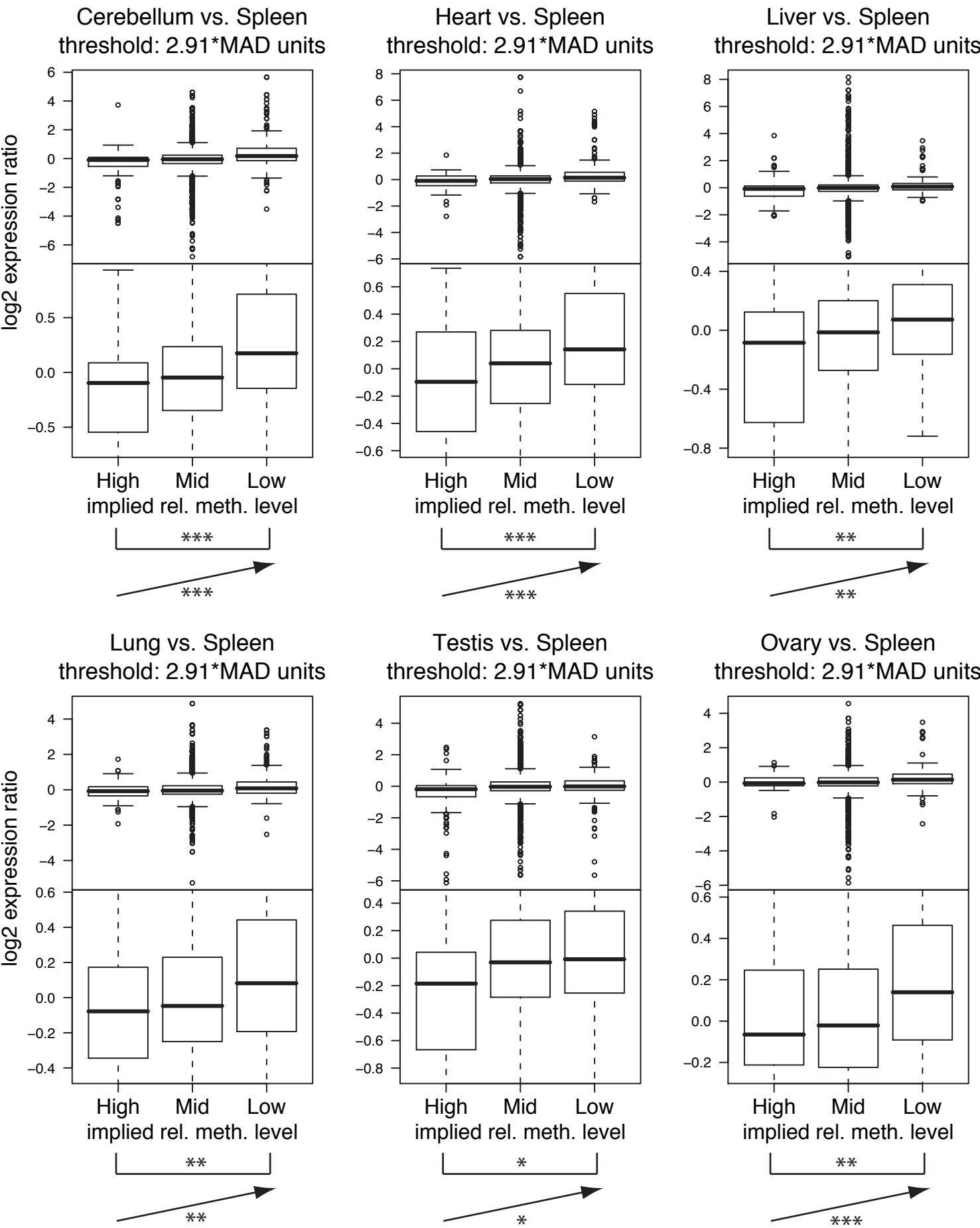

Supplement: Additional File 10 — Figure S9. Expression ratios tend to be higher for genes whose 5' ends fall in regions of lower methylation (that is, in bacterial artificial chromosomes (BACs) with higher methylation array ratios) than those in regions of higher methylation. Data are presented as in Figure 7, but here BACs are classified in the 'High' and 'Low' categories using a less conservative methylation array ratio threshold. This less conservative threshold is defined as 2.91 * median absolute deviation (MAD) units below and above the median value. At the 2.91 * MAD threshold, we expect approximately 2.5% of BACs on the array to fall into each of the 'High' and 'Low' categories purely by chance (that is, an overall false-positive rate of 5% of array BACs). Figure 7 conservatively uses 4.88 * MAD, such that the false-positive rate is expected to be only around 0.1% of the BACs on the array. The increased number of BACs in the 'High' and 'Low' categories using this less conservative approach likely gives the statistical tests greater power, explaining the greater significance of most P values shown here as compared with Figure 7. [file 1756-8935-2-7-S10.pdf]

Figure S10

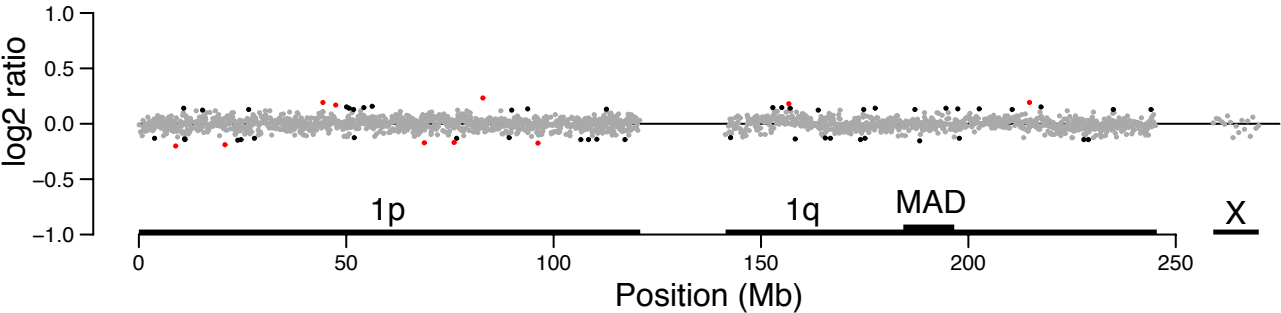

Supplement: Additional File 11 — Figure S10. Methylation profile in which two independently processed aliquots of DNA isolated from medulla tissue from the same female donor are directly compared. The log2 ratios for chromosome 1 clones are indicated at their relative genomic position, with the large gap representing the centromere and pericentromeric repeats in 1p11–1q21. The horizontal scale for the X chromosome is compressed; the 17 chromosome X clones on the array are distributed over 110 Mb. Clones highlighted in black or red have log2 ratios that deviate from the overall median, set here to 0, in either direction by >4.88 median absolute deviation (MAD) units, where MAD units are calculated using the data for the clones in the region of 1q marked 'MAD', which showed the least biological variation within and across experiments. Clones highlighted in red deviate from the overall median by ± 4.88 MAD, calculated using all values. [file 1756-8935-2-7-S11.pdf]
